# Practical guidelines for exercise prescription in different clinical populations

**DOI:** 10.3389/fspor.2026.1649549

**Published:** 2026-03-02

**Authors:** Klara Komici, Antonio Bianco, Alessandra Cuomo, Roberto Bianco, Maddalena Illario, Germano Guerra, Antonio Paoli, Federico Schena, Guido Iaccarino

**Affiliations:** 1 Department of Medicine and Health Sciences, University of Molise, Campobasso, Italy; 2 Department of Public Health, University of Naples Federico II, Naples, Italy; 3 Department of Clinical Medicine and Surgery, University of Naples Federico II, Naples, Italy; 4 University of Naples Federico II, Naples, Italy; 5 Department of Biomedical Sciences, University of Padova, Padova, Italy; 6 Department of Neurosciences, Biomedicine and Movement Sciences, University of Verona, Verona, Italy

**Keywords:** ageing, chronic diseases, exercise prescription, exercise training, physical activity, physical capacity

## Abstract

Exercise training represents a cornerstone therapeutic intervention for managing chronic health conditions, yet its practical implementation in clinical settings remains suboptimal due to challenges in individualization and safety considerations across diverse patient populations. This narrative review provides practical guidelines for exercise prescription in outpatients with chronic conditions, grounded in recent international recommendations and current scientific evidence. Practical considerations for exercise prescription are addressed across various chronic conditions including cardiovascular disease, heart failure, diabetes, and cancer, with particular emphasis on pre-exercise evaluation in patients with hypertension-related organ damage, decompensated heart failure, and frailty. Exercise prescription should be personalized and adapted to individual health status through gradual and progressive incremental physical activity programs designed to optimize health outcomes while minimizing risks. Social, psychological, environmental factors, and technology integration represent important determinants of adherence that warrant systematic consideration. The successful translation of exercise prescriptions into effective training programs requires dedicated clinical facilities staffed by specialized professionals who can bridge the gap between prescription and implementation.

## Key points

- These practical guidelines include considerations for exercise prescription in outpatients with cardiovascular risk factors, chronic coronary disease, heart failure and cancers.
- The pre-exercise evaluation is described with special consideration to conditions which may represent contraindications to exercise prescription.
- Frequency, intensity, time and type of exercise are summarized to help clinicians in the specific prescription of exercise program.

## 1 Introduction

The World Health Organization (WHO) has provided guidelines on physical activity and sedentary behavior for people of different age groups, including those with disabilities and chronic conditions (1). Physical activity offers unquestionable health benefits for people of all abilities and ages. A target range of 150–300 min of moderate intensity or 75–150 min of vigorous intensity per week are necessary for a maximal health reduction of negative health outcomes (2). Additionally, muscle strengthening and balance training applies to all adults as part of the multi-component physical activity. Sedentary behavior should be minimized and replaced with physical activity of any intensity for all age groups (2).

Given the positive impact of physical activity and exercise on energetic metabolism and multiple systems and organs, such as the cardiovascular system, its prescription in conditions characterized by increased cardiovascular risk such as diabetes, arterial hypertension, dyslipidemia and obesity is considered a cornerstone of treatment (3). This point holds true also in patients with established chronic conditions such as chronic coronary syndrome and heart failure where exercise training has been shown to reduce cardiovascular events, hospitalizations and overall mortality (4–6), as well as improved left ventricular remodeling, enhanced quality of life, and increased event-free survival (5, 7).

Exercise is also crucial for patients with cancer: an inverse correlation between perioperative complications and physical activity has been reported. A significant improvement of survival in patients with breast and colon cancer compared to sedentary patients has been reported (8, 9). Collectively, the benefits of exercise and physical activity extend beyond health, positively influencing social, economic, and environmental fields.

Despite the unanimous consent regarding the pivotal role of physical activity and exercise prescription for clinical conditions, guidelines on prescription methodology are scarce. In an attempt to provide insights on the methodology for physical activity prescription, American College of Sports Medicine (ACSM) has developed a comprehensive set of guidelines for exercise testing and prescription, now in its 12th edition (10). These guidelines focus on health screening before starting regular physical activity and clinical applicability of exercise. They represent a crucial resource for the healthcare providers in prescribing exercise in everyday clinical practice. Nevertheless, knowledge and training about exercise prescription among healthcare professionals are scarce. Indeed, in a survey of the American Medical Society of Sports Medicine including directors and fellows of Sports Medicine 48% of the fellows do not write exercise prescription, 63% of the fellows reported that they did not receive knowledge regarding how to write exercise prescription (11). Furthermore, a significant disagreement with current guidelines and a significant inter-clinician variance in exercise prescription has been reported. Cardiac rehabilitation clinicians from European countries resulted heterogeneous regarding intensity, frequency, session duration, program duration and strength training program prescribed (12). Data from an Italian standardized survey submitted to general practitioners (GPs), reveal that only 24% of GPs perform or prescribe physical capacity evaluations. Despite 93% of the GPs regularly prescribed physical activity and exercise, only 15% provided recommendations in writing (13). A prospective observational survey focused on exercise prescription in cardiovascular diseases patients including Belgian physiotherapists found that exercise prescription was often in discordance with European recommendations and varied widely (14).

Motivating people to participate is also challenging. Adherence to physical activity recommendations in Norwegian adolescents during 2019–2021 resulted low (15). On the contrary, in patients with chronic conditions such as diabetes, cancer and cardiovascular diseases, the adherence rate resulted instead higher: 65%, 80% and 90% respectively (16).

Given the well-established health benefits of physical activity and exercise (17), and in alignment with the Global Action Plan on Physical Activity to promote adherence to exercise training, we summarize practical guidelines for exercise prescription in outpatients with cardiovascular risk factors, chronic coronary disease, heart failure, and cancers. We chose the form of a narrative review to synthesize current evidence and international recommendations to provide guidance for exercise prescription in clinical practice. This review describes exercise prescription protocols, pre-exercise evaluation, and the contraindications and safety considerations for exercise. Furthermore, the review focuses on translating scientific evidence into a practical tool helpful for all healthcare providers involved in exercise prescription for outpatients with chronic conditions.

We initially describe the pre-exercise evaluation, subsequently, we describe the exercise prescription and also the contraindications and safety aspects of physical exercise in these outpatients.

For the purposes of this narrative review an article search was performed on MEDLINE/PubMed using combinations of the following terms: “exercise training”, “physical activity”, “arterial hypertension”, “diabetes”, “heart failure”, “dyslipidemia” “cancers” and “frailty”. The selection of literature prioritized recent international guidelines, meta-analyses, randomized controlled trials, and studies that inform current clinical practice.

## 2 Pre-exercise evaluation

The benefits of regular, moderate-intensity exercise (18) far outweigh its rare side effects. The pre-participation health screening process should not be a burden on the physician or prevent patients from initiating this activity. Asymptomatic patients can use validated questionnaires such as the American Heart Association (AHA)/American College of Sports Medicine (ACSM) Health/Fitness Facility Pre-participation Questionnaire or the revised Physical Activity Readiness Questionnaire (PAR-Q) to determine whether their risk is such that they should consult a physician before starting physical activity. Other validated physical activity questionnaires across European Union Countries are: International Physical Activity Questionnaire (IPAQ-SF), Global Physical Activity Questionnaire (GPAQ) (19), and European Health Interview Survey-Physical Activity Questionnaire (EHIS-PAQ). These tools have been recommended for assessing moderate to vigorous physical activity, although they require time to administer (18). The Physical Activity Scale for the Older Adult (PASE) is a self-administrated questionnaire, which includes 10 items. It is easy to apply and time saving, which has demonstrated high reliability and validity in different clinical settings (20). Furthermore, the predictive impact of PASE scale has been demonstrated widely (21).

Alternatively, for such patients, a history of cardiac, pulmonary, and metabolic risk factors, physical activity level, and the detection of signs and symptoms during a routine medical examination is enough.

Information on medical history and CV risk factors is an important part of the pre-exercise evaluation. These information aid in:

(a) identifying medical conditions that may contraindicate exercise training
(b) assessing individual's current level of physical activity and/or sedentary behavior
(c) tailoring exercise prescriptions to specific and individual health conditions
(d) establishing the appropriate exercise intensity.

The medical history should include information regarding the presence of conditions such as arterial hypertension, dyslipidemia, coronary artery disease, arrhythmias, congenital heart diseases, heart failure, implantation of pacemaker and implantable cardiac defibrillator, diabetes, peripheral artery diseases cerebrovascular diseases, diabetes and renal diseases. Other relevant Conditions including smoking, COPD, Dementia, Parkinson's Diseases, Arthrosis, Osteoporosis, and chronic pain. In addition, information regarding current therapy is necessary.

Chest pain or other angina equivalent, fatigue, shortness of breath, palpitations, heart murmur, ankle edema, and intermittent claudication are major signs and symptoms suggesting a possible CV, metabolic or renal diseases and should be, therefore, further evaluated.

According to ACSM, participants are classified as exercisers if a planned and structured physical activity is performed for at least 30 min on three days or more during the past three months (10). If these conditions are not fulfilled, the participants are classified as not exercisers.

### 2.1 Contraindications to exercise prescription and special considerations

#### 2.1.1 Arterial hypertension

Individuals affected by arterial hypertension should not be engaged in any exercise program in the case of:

- stage 2 arterial hypertension systolic blood pressure (SBP) ≥160 mmHg diastolic blood pressure (DBP) ≥ mmHg (22);
- organ damage relative to hypertension as: ischemic stroke/ TIA, intracranial hemorrhage, retinopathy, cognitive decline, left ventricular hypertrophy, chronic kidney disease.

These conditions require further evaluations for instance: 12 lead resting ECG, echocardiography, serum creatinine, eGFR, albuminuria, arterial color doppler echography. Fundoscopy, evaluation of cognitive performance and brain imaging may be useful for specific population and clinical context. Cardiovascular risk factors often cluster with each other and estimation of the total cardiovascular risk is of great importance for the correct management of hypertensive patients. European Guidelines on CVD prevention have recommended use of the Systematic COronary Risk Evaluation (SCORE2) system in individuals of age 40–69, and SCORE2-OP for individuals 70–89 years old, which estimates the 10year risk of a first fatal atherosclerotic event (23, 24). Once the blood pressure control is adequate exercise prescription may be programmed. The current European Guidelines recommends an office BP target <130/80 mmHg in patients 18–64 years, <140/80 mmHg in patients 65–79 years, SBP range 140–150 in patients over 80 years old, or 65–79 of age with isolated systolic hypertension (25).

#### 2.1.2 Dyslipidemia, overweight, obesity and diabetes

No contraindications for exercise prescription are present for individuals affected by dyslipidemia, overweight and obesity. However, caution during pre-exercise evaluation regarding underlying cardiovascular risk factors and other chronic conditions as: hypertension, diabetes, metabolic syndrome. In obese patients the presence of orthopedic and musculoskeletal conditions may require the need for low initial workload and use of arm or leg ergometry. Furthermore, in individuals taking lipid lowering drugs the presences of muscle weakness and myalgia should be detected and monitored during exercise training.

Diabetes mellitus is a chronic endocrine disorder characterized by hyperglycemia resulting from defects in insulin secretion, insulin action, or both (26). There are two basic types of diabetes, insulin dependent (DM1) and non–insulin dependent (DM2). Symptoms of hyperglycemia include polyuria, polydipsia, weight loss, sometimes polyphagia and blurred vision. The presence of chronic hyperglycemia typical of diabetes is associated with long-term damage, dysfunction, and failure of various organs, especially the eyes, kidneys, nerves, heart, and blood vessels.

Individuals with severe diabetic retinopathy should avoid aerobic or resistance exercise of vigorous intensity. Hypoglycemia, blunted heart rate response and post- exercise hypotension are concerns in diabetic patients during exercise training. Pre-exercise optimal glycemia level should be 90–250 mg/dL and strict monitoring of glycemia level, heart rate and blood pressure during exercise may be necessary.

#### 2.1.3 Heart failure

Clinical instability, decompensated Heart Failure (HF), worsening of exercise intolerance are conditions that exclude HF patients from exercise prescription (27). Stable and optimal guideline-directed medical therapy patients are eligible for exercise prescription based on recommendations of The American Heart Association (AHA) and American College of Cardiology (ACC) guidelines (28). Stable patients are defined as patients who have not undergone major unplanned cardiovascular hospitalization or procedures in the last 6 weeks, or planned hospitalizations and procedures during the last 6 months.

#### 2.1.4 Cancer

Post-operative exercise timing should be individualized based on surgical procedure, extent of resection, patient health status, and wound healing. The 2019 ACSM International Multidisciplinary Roundtable emphasizes that cancer survivors should avoid inactivity and return to normal daily activities as quickly as possible after surgery (29).

Surgery-specific timelines guide safe and effective exercise initiation:

Breast cancer surgery: Range of motion exercises begin 1–2 days post-surgery, with evidence from a 2024 JAMA Surgery RCT demonstrating that early exercise implementation significantly improves functional recovery without increasing adverse events. Full upper body exercise typically resumes at 4–6 weeks (30).

Colorectal cancer surgery: Enhanced Recovery After Surgery (ERAS) protocols emphasize enforced mobilization within 24 h post-surgery, with structured exercise programs initiated at 2–4 weeks and full programs at 4–6 weeks for laparoscopic procedures (31).

Lung cancer surgery: Pulmonary rehabilitation begins day of surgery (unless complications occur), with supervised programs continuing during hospitalization and transitioning to outpatient rehabilitation at 2–4 weeks. Recovery timelines vary by surgical approach (VATS vs. open thoracotomy) and extent of resection (32).

Prostate cancer surgery: Exercise begins immediately post-operatively, with progressive resistance training initiated at 2–4 weeks. Pelvic floor exercises start as soon as the catheter is removed (33).

Progression follows a phased approach from immediate post-operative mobilization through early recovery (weeks 1–4), progressive reconditioning (weeks 4–12), and maintenance (week 12+), with modifications for individual patient factors and treatment-related complications

Contraindications to exercise are: hemoglobin concentration <8 g/dL; oxygen saturation on room air <88%, white blood cell number <2,000/mm^3^; neutropenia <1,500/mm^3^. Exercise should be avoided if symptoms such as: syncope, wheezing, shortness of breath, claudication are present. The presence of upper extremity lymphedema, bone metastasis, ostomy and neuropathy should be evaluated with caution since resistance training should start with low resistance and progress slowly, loading of muscle that are proximal to metastatic lessons should be avoided, systematic evaluation for falls risk may be necessary. Risk of fractures should be considered in patients with bone metastasis, prostate cancer, diagnosis of osteoporosis, and treatment with hormonal therapy. Swimming and water sports should not be prescribed in cancer patients undergoing radiotherapy, patients with central lines, and ostomies.

### 2.2 Evaluation of physical capacity

All patients should undergo preparticipation health screening, regardless of age. The screening procedure should be simple and easy to administer and inexpensive. It includes a medical history, full physical examination, physical capacity, strength, flexibility, balance, and body composition.

#### 2.2.1 Cardiopulmonary exercise testing to assess physical capacity

Evaluation of physical capacity is closely aligned to cardio-respiratory performance, disease prevention, skills related components and dependency living status. By measuring physical capacity physicians and healthcare providers can:

(a) evaluate baseline health status before exercise prescription
(b) provide prescription of tailored exercise programs related to specific populations and health- related conditions
(c) monitor the effect of exercise on cardio-respiratory performance and muscle strength
(d) motivate and educate

Cardio-Pulmonary Exercise Testing (CPET) is considered the gold standard for physical capacity evaluation, providing a comprehensive global assessment of the cardiovascular, respiratory, metabolic, and skeletal muscle systems' responses to exercise. Clinical applications of CPET include: evaluation of exercise tolerance and unexplained dyspnea, assessment of patients with cardiovascular or respiratory disease, preoperative risk evaluation, lung and heart transplant candidate evaluation, prescription of exercise and rehabilitation programs for cardiorespiratory diseases (34, 35). CPET systems contain gas analyzers and flow meters which allow breath to breath measurements of O2 uptake (VO2), exhaled CO2 (VCO2) and ventilation. Maximal O2 uptake (VO2 max) is the highest VO2 averaged over at least 20 s, achieved at the maximal effort during incremental exercise. VO2 max describes the maximal amount of energy obtainable by aerobic metabolism and the ability of cardiorespiratory system to deliver O2 to tissues. Age, sex, body size, genetic factors and exercise are considered contributors to individual VO2 max variation (36)*.* Despite genetic factors determine about 50% of VO2max in sedentary patients, exercise training has been reported to improve cardio-respiratory fitness (37). Of note a recent study indicated no sex-specific differences for cardiorespiratory Fitness and Performance Adaptations to High-Intensity Interval Training (38). Moreover, VO₂max and dysfunctional breathing during exercise have been shown to influence prognosis across various clinical settings (39, 40). During incremental exercise there is a point where energy requirement is higher than oxygen resources and anaerobic mechanism is activated. Consequently, blood lactate increases above the baseline level at a steeper way. The metabolic increase due to the rate of lactate production is higher than disposal capacity. Products of lactic acids are buffered by bicarbonates yielding and excess CO2 production and ventilation increases more steeply relative to VO2 increase. The first ventilatory threshold (VT) is the point where ventilatory equivalent for VO2 becomes steeper, while the ventilatory equivalent for VCO2 is stable (41). VT is expressed as percentage of VO2 max or as VO2 mL/kg/min, and usually occurs at 40%–70% of the VO2max in healthy subjects. It has been reported that exercise training increases the VO2 at VT up to 25% (42). With increasing exercise intensity there is an exponential increase in blood lactate concentration, excessive VCO2, hyperventilation and VO2 increase remains linear. This second breakpoint in ventilation response to exercise (second VT) is identified as respiratory compensation point (RCP), which is identifiable by nadir of the VE/VCO2 to workload curve (43). Other important parameters of CPET include: breathing reserve (BR), which is defined as one minus the ratio of maximal VE during exercise to estimated maximum voluntary ventilation, and ventilatory efficiency and ventilatory efficiency (VE/VCO2 slope) which derives from the ratio of pulmonary ventilation to VCO2 production, and is considered to reflect right ventricular-pulmonary vascular function during exercise (44).

The physiological response to exercise is characterized by the first VT, RCP and VO2max, which allow the identification of three intensity zones. For accurate exercise prescription heart rate and workload to each appropriate zone should be used for exercise prescription. Zone 1 represents light to moderate intensity exercise, zone 2 moderate to high intensity training includes workloads between the first VT and RCP. Zone 3 represents workloads above the CP that result in VO2max at exhaustion and constitute high to sever exercise intensity (43).

In patients with HF CPET parameters are characterized by reduced VO2max, first VT below 40% of the VO2 max and wide BR, while in patients with COPD, BR is reduced and ventilatory inefficiency is pronounced (38, 39, 41, 45, 46). Table 1 summarizes CPET parameters described above.

**Table 1:** Parameters extrapolated from CPET.

Parameter	Definition	Physiological Meaning	Clinical Relevance
VO₂max	Highest VO₂ during maximal incremental exercise	Maximal aerobic capacity and ability of the cardiorespiratory system to deliver O₂ to tissues	Influenced by age, sex, body size, genetics and training; improves with exercise
Anaerobic threshold	Point where energy demand exceeds O₂ availability	Lactate production exceeds clearance	Leads to increased VCO₂ and ventilatory response
First Ventilatory Threshold (VT1)	Exercise intensity where VE/VO₂ increases while VE/VCO₂ remains stable	Onset of significant anaerobic contribution	Occurs at ∼40%–70% of VO₂max in healthy subjects; increases with training (+25%)
Second Ventilatory Threshold/RCP	Respiratory compensation for metabolic acidosis	Exponential rise in lactate and VCO₂ with hyperventilation	Identified by nadir of VE/VCO₂ vs workload curve
VO₂ response to exercise	VO2 consumption during exercise	Linear increase until exhaustion. Reflects progressive metabolic demand	Plateau indicates attainment of VO₂max
Breathing Reserve (BR)	1—(maximal exercise VE/estimated MVV)	Remaining ventilatory capacity	Reduced in respiratory diseases
Ventilatory efficiency (VE/VCO₂ slope)	Ratio of ventilation to CO₂ production	Indicator of ventilation–perfusion matching	Reflects right ventricular–pulmonary vascular function

Despite its established clinical utility, CPET remains significantly underutilized in contemporary clinical practice. Multiple barriers contribute to this limited adoption, including the technical complexity of the test, insufficient training among healthcare specialists in CPET interpretation, and poor understanding of its parameters among referring physicians. Operational challenges further compound the issue, with equipment costs and limited availability. Addressing these barriers is essential to enhance CPET utilization and optimize patient care. Of interest application of artificial intelligence in CPET interpretation may provide benefits, including improved diagnostic accuracy, reduced interobserver variability, and expedited decision-making (47).

Although CPET represents the only test for multiparametric assessment of functional capability, limitations due to dedicated equipment, personnel qualifications and complex execution have led to the development of simpler functional tests using surrogate parameters to assess functional capacity.

The following paragraphs illustrate such tests.

**Figure 1:**
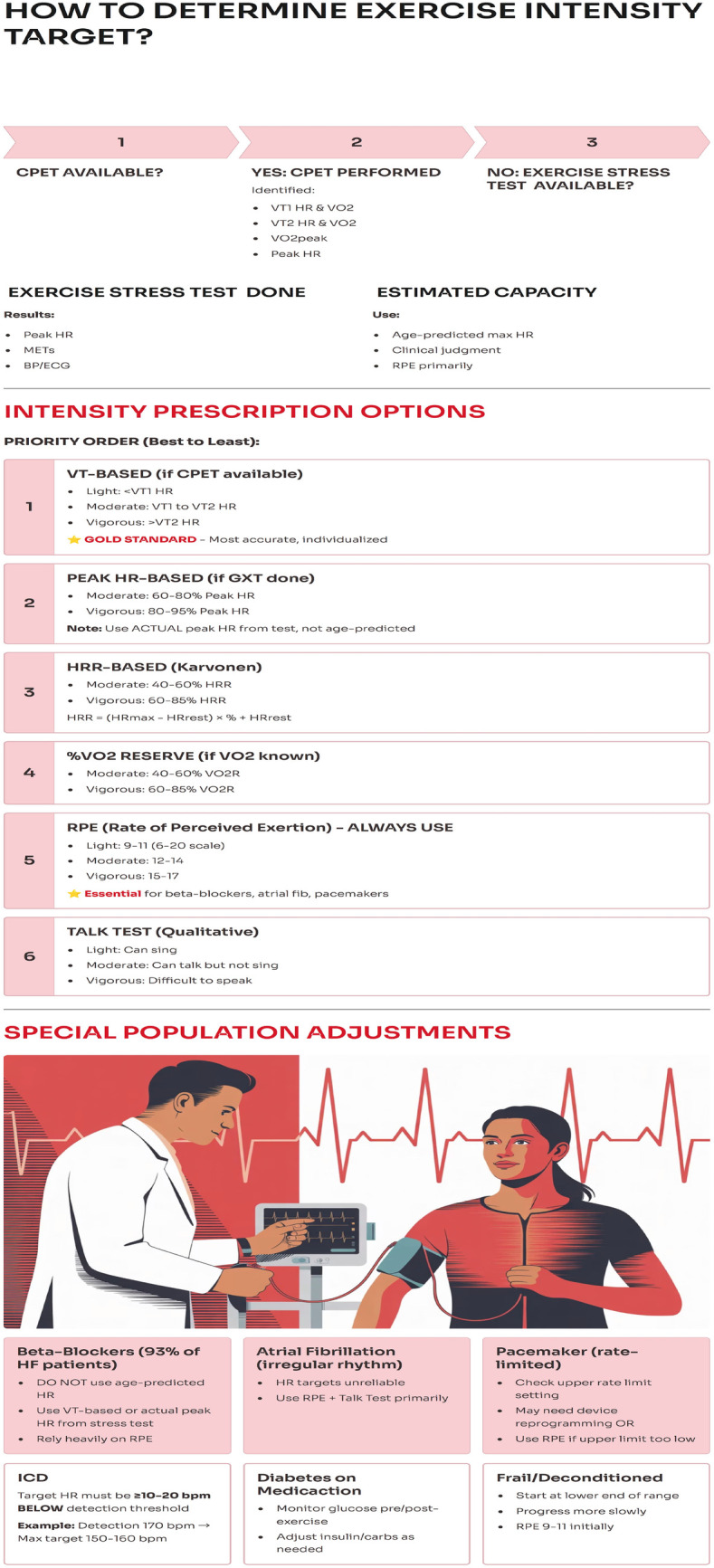
Graphical Flowchart for exercise intensity prescription. Created with Gamma AI.

Also, a graphical flowchart in Figure 1 summarizes exercise intensity evaluation based on CPET and other tests.

#### 2.2.2 Electrocardiography (ECG) stress testing

In assessing functional capacity, stress testing is used due to its simplicity and cost-effectiveness. The most used ergometric parameters for this purpose are heart rate and exercise tolerance.

In healthy subjects, exercise intensity is defined as the percentage of maximum heart rate using the formula 220-age, or in subjects over 50–60 years of age, the Tanaka formula (208–0.7×age) (48). In cardiac patients, it is preferable to use the real heart rate, that is, the heart rate reached during an exercise test under optimized pharmacological therapy, or the heart rate reserve, which is given by the difference between the maximum heart rate reached during the exercise test and the heart rate at rest. Exercise intensity can also be defined by the workload expressed in watts if using a cycle ergometer or in incline speed if using a treadmill. A parameter associated with good health and a reduced risk of death from cardiovascular events (49) is a rapid decrease in heart rate (30–40 bpm) in the recovery phase of the exercise test (first minute).

In this context is also pivotal to underline the role of metabolic equivalents (METs) in the assessment of activity status of patients. In particular, METs are a measurement of physical activity, defined as the ratio between the energy expended during any given activity compared to the energy at rest, being 1-MET the energy required to sit still (corresponding to 3.5 mL O2 per kg per min of activity) (50).

Age-appropriate exercise time is associated with a favorable prognosis, and a 1-MET increase in exercise tolerance, for males, results in a 12% increase in 6-year survival (51). Furthermore, it has been demonstrated that patients that present a peak exercise capacity inferior to 4.3 METs present higher risk of mortality compared to those reaching 4.3–6.3 or >6.3 METs, with hazard ratios for total mortality declining from 1.0 to 0.66 and 0.45 (52, 53).

#### 2.2.3 Other exercise functional capacity testing

Other tests that allow to evaluate exercise capacity and define VO2 Max indirectly are: the Bruce and Balke test, the indirect Mader test, the 6-min walking test and the 12-min Cooper test.

The Bruce and Balke test is performed on a cycle ergometer or treadmill and calculates VO2 max indirectly through specific tables. This test is highly versatile and can be applied to athletes, healthy subjects, and those with heart disease by varying the amount and method of increasing workload. Exercise capacity and intensity can be expressed using the “relative percentage method,” i.e., using the percentage of maximum heart rate (%HR Max) or reserve (%HR Reserve), or the percentage of maximum oxygen consumption (%VO2 Max) or reserve (%VO2 Max Reserve). %VO2 Max and %HR Max exhibit considerable interindividual variation and may not ensure adequate control of exercise intensity, resulting in some individuals who respond well and others who do not respond to chronic exercise training (54).

Cardiac reserve percentage has been adopted in the past as the gold standard for the indirect determination of exercise intensity but, in recent years, the use of heart rate as an index of metabolic intensity has been questioned, since, during prolonged and constant exercise (longer than 10 min) heart rate and oxygen consumption diverge over time as a result of a slow increase in heart rate independent of metabolism (54, 55).

The modified Mader test allows to identify the training load and the target heart rate during training by measuring lactic acid in capillary blood during a stress test.

The 6-min walking test (56) and the 12-min Cooper test (57) are the most widely used clinically to indirectly calculate VO2 max and the perception of physical exertion. In the absence of monitoring systems, exercise intensity can be defined by the sensation of fatigue during exertion using the Borg scale, a 15-point scale ranging from 6 to 20, or by informally applying the Talk Test. Moderate activity can be performed at a pace that allows for fairly comfortable speaking despite increased ventilation, while vigorous activity corresponds to an intensity that makes speaking difficult due to the increased rate and depth of breathing.

#### 2.2.4 Major muscular group strength

##### 2.2.4.1 Upper limb strength

Hand grip strength (HGS) is a simple and low- cost method in the measurement of muscle strength and screening of sarcopenia (58). Accurate measurement of muscle strength requires the use of a calibrated handheld dynamometer with interpretive data from appropriate reference populations. European Working Group on Sarcopenia in Older People (EWGSOP2) indicates as cut-off point for sarcopenia diagnosis less than 27 kg in men and less than 16 in women (58). In addition, HGS has been reported as a powerful method in the prediction of survival and healthy ageing (59–61).

##### 2.2.4.2 Lower limb strength

Evidence supports that HGS is indicative of overall muscle strength, including also lower limb strength (62). However, assessment of lower limb strength may be useful in the population where HGS is not feasible or in the context of exercise programs which may expose patients to knee ligament injury (62, 63). Nicholas Manual Muscle Tester is a hand-held device for quantification of isometric muscle strength including hip abduction, flexion and knee extension (64). Measurement of lower limb muscle strength with hand-held dynamometer which records the peak force required to break an isometric contraction has been suggested as a valid method in quantifying age-related muscle dysfunction (65). The Chair and Stand Test is used for the measurement of quadriceps muscle groups and measures the time needed to rise five times from a seated position without using arms. Another version of this test is the counts of how many times the patients can rise and sit during 30 s intervals. EWGSOP2 indicates that more than 15 s for 5 rises as a cut off point for sarcopenia (58, 66).

#### 2.2.5 Flexibility evaluation

Flexibility is the intrinsic property of muscles and connective tissue to determine the range of motion (ROM), achievable without injury at a group of joints or a single joint (67). Shoulder stretch, trunk lift, sit and reach test, bilateral sit-and reach test and back-saver sit and reach tests are field tests applied in the evaluation of flexibility (67). Passive straight leg test raise test and passive knee extension in the isokinetic dynamometer are also methods applied in ROM assessment (68).

#### 2.2.6 Balance evaluation

Balance impairment is common among older adult adults and in patients with multiple comorbidities. Therefore, before the prescription of therapeutic exercise evaluation of balance and risk of falls is essential in special populations. Visual, vestibular, audiologic and symptoms stimulation tests are applied in the ambulatory settings for the clinical assessment of balance (69). Functional performance tests evaluate the movements and postural activities which occur in the occur during everyday life. The timed up-and-go evaluates dynamic balance and measures the time taken by the seated individual to stand up from a standard armchair, walk a distance of 3 meters, turn, walk back to the chair and sit down. Community dwelling is considered unsafe for persons who take >20 s to complete the test (70).

Another validated method for balance assessment in older adult is the Performance-Oriented Mobility Assessment (POMA) which consists of balance and gait subscales. The POMA takes only about 10 min to complete and can fit into most clinical contexts (71).

#### 2.2.7 Body composition evaluation

Body composition describes and qualifies various elements within human body such as fat mass, total body water, muscle mass and bone mass. Anthropometry measures such as: simple measurement of subcutaneous fat body weight, height, calculation of body mass index (BMI) and waist circumference are important not only for the detection of overweight and obesity, but also to monitor the health-related changes in body composition associated to ageing process. Other simple measures which have demonstrated a valuable role in the identification of cardio-vascular risk and physical capacity as well are waist to hip ratio and a body shape index (72–74). Dual-energy x-ray absorptiometry (DXA), magnetic resonance, and computer tomography are methods characterized by high accuracy, reliable and repeatable for the evaluation of both muscle mass, fat mass regional and total evaluation as well (75). However, these methods are characterized by high costs and radiation exposure. Bioelectrical Impedance Analysis (BIA) is a quick and not invasive method, which measures the impedance (Z) to the flow of an alternating current, which is directly related to body's fluid content and its distribution among intra and extracellular spaces, allowing to estimate hydration status, nutritional status (via bioelectrical impedance vector analysis, BIVA), and prognosis (76, 77).

### 2.3 Further assessment in specific populations

#### 2.3.1 Short physical performance battery (SPPB)

The SPPB is a three-part physical function test with excellent reliability, validity, and clinical applicability for the physical capacity of older adult, risk of falls and overall survival (78, 79). SPPB consists of 3 components: (1) 4-m usual walk (2) five-repetition chair stand without using one's arms, and (3) progressive test of standing balance. Study personnel demonstrated each component ahead of participant testing. Total SPPB scores ranges between 4 and −12, with 4–6 representing low, 7–9 middle, and 10–12 best performances (78). Table 2 summarizes use of functional capacity evaluation methods.

**Table 2:** Summary of the functional capacity evaluation methods.

Test	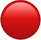 RED FLAG (Defer Exercise/Urgent Evaluation)	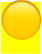 YELLOW FLAG (Modify Exercise/Close Monitoring)	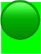 GREEN FLAG (Safe to Proceed/Progress)
6-Minute Walk Test (6MWT)	Distance <150 m (severe impairment) Unable to complete test (stops due to symptoms) Significant desaturation (O2 sat <88% on room air) Severe dyspnea/chest pain during test	Distance 150–300 m (moderate impairment) Completed but with symptoms (dyspnea, leg pain) Oxygen saturation 88–92% No improvement or decline >30 m (MCID) from baseline after 4–12 weeks of exercise	Distance >300 m (mild impairment or normal) Completed without significant symptoms O2 sat >92% throughout Improvement ≥30 m (MCID) after exercise program indicates meaningful benefit
Handgrip Strength (HGS)	< 16 kg (women) or <27 kg (men) AND declining over time Severe sarcopenia with functional impairment Combined with very low SPPB (<4)	16–20 kg (women) or 27–32 kg (men)—borderline sarcopenia No improvement or decline >5–6 kg (MCID) after resistance training program	≥20 kg (women) or ≥32 kg (men)—normal Improvement ≥5–6 kg (MCID) after resistance training indicates meaningful strength gain Safe to progress resistance training
Timed Up and Go (TUG)	>20 s (very high fall risk) Unable to complete without assistance Near-fall or loss of balance during test Combined with history of recent falls	13.5–20 s (moderate-high fall risk) Completed but slow, unsteady No improvement or decline >3.5 s (substantial change) after balance training	<13.5 s (low fall risk) Completed smoothly, steady Improvement ≥3.5 s (substantial change) or ≥2.5 s (MCID) after balance training indicates meaningful fall risk reduction
SPPB Score	0–3 points (high risk—severe functional impairment, very high fall/disability risk) Score declining over time	4–6 points (moderate risk—moderate functional impairment) No improvement or decline >1 point (MCID) after exercise	7–12 points (low risk—mild or no impairment) Improvement ≥1 point (MCID) or ≥2 points (substantial change) after exercise indicates meaningful functional gain

#### 2.3.2 Frailty evaluation

Frailty is a clinical condition characterized by vulnerability, associated with loss of physiological reserves and declining function of multiple physiological systems (80–82). Frailty has tremendous effects on dependence, disability, hospitalizations, mortality, hospitalization, and significant healthcare cost (83). Two main models are used to define frailty:

(a) the physical phenotype of frailty which considers muscle strength, gait speed, physical inactivity, weight loss and exhaustion (84);
(b) accumulation of deficit model which includes the accumulation of multiple deficits such signs, symptoms, comorbidities, disabilities and abnormal laboratory values (85).

For the screening of frailty may be useful the phenotype model and to determine the severity of frailty the multidimensional models. Fried's frailty phenotype (FP) represents a physical frailty model and stands as one of the most widely utilized frailty assessment tools for prognostic purposes. This model encompasses five key criteria: unintentional weight loss of 4.5 kg or more within the preceding year, reduced handgrip strength, self-reported exhaustion, decreased walking speed, and diminished physical activity levels. The presence of three or more criteria indicates frailty, while individuals meeting one or two criteria are classified as prefrail. The Frailty Index (FI), based on deficit accumulation, incorporates a comprehensive range of health parameters including symptoms, signs, functional disabilities, and comorbidities. This model calculates frailty by dividing the number of identified health deficits by the total variables assessed, with higher deficit counts indicating greater frailty severity. The FI derived from comprehensive geriatric assessment encompasses functional, nutritional, cognitive, and psychological evaluations, demonstrating strong correlation with the deficit accumulation-based FI (85). Additional validated frailty measurement instruments include the Tilburg Frailty Indicator, Edmonton Frailty Scale, Clinical Frailty Scale, Study of Osteoporotic Fractures Frailty Index, and Multidimensional Prognostic Index. These tools, derived from the primary frailty models, have undergone validation across diverse populations and clinical contexts.

### 2.4 General principles of exercise prescription

The fundamental components of exercise prescription, commonly known as FITT principles involve F*requency*, I*ntensity*, T*ime* and T*ype of exercise* (17).

F) The **frequency** of exercise training refers to the number of days per week dedicated to exercise, optimally 3–5 days/ week.
  I) Exercise **intensity** can be estimated with various methods including heart rate (HR), oxygen uptake (VO_2_), metabolic equivalents (METs) or perceived exertion rate (RPE) such as 6–20 scale or 0–10 scale. Heart rate reserve (HRR) is calculated by the difference between predicted maximal HR (HR max) and resting HR. Considering %VO_2max_ evaluated with CPET, 37%–45% of VO_2max_ corresponds to light intensity, 46%–63% of VO_2max_ moderate intensity and 64%–90% vigorous intensity. RPE of 12–13 corresponds to moderate intensity, and 14–17 vigorous intensity.
T) Exercise **time** is the amount of time spend in exercise training, usually expressed as minutes/day (17).
T) Exercise **type** includes a variety of exercise modes which are part of the aerobic, resistance, or flexibility exercise training. When prescribing exercises, volume and progression of exercise program should be taken in consideration. The volume is the product of frequency, exercise intensity and the duration of exercise sessions. Exercise volume (dose) is the total amount of exercise performed. It is given by the product of the frequency, the time and the intensity of the exercise.

The progression is the increase of volume to be scheduled according to the achievement of training.

Endurance exercise training defines any physical activity that uses large muscle groups, with cyclic and repeated movements and relies on aerobic metabolism to produce energy. Examples of endurance exercise training are: walking, dancing, swimming, running, cycling (17, 86). Resistance exercise training is characterized by any physical activity that requires force against a resistance. Resistance exercise improves muscular strength, hypertrophy, local muscle endurance and power. Examples of resistance training are: free weights, machines, body weight, and elastic bands (17, 86).

Flexibility exercise training is the ability to move through a joint's range of motion. Type of flexibility exercise training include ballistic methods, bouncing stretches, dynamic or slow movement stretching, active and passive stretching, proprioceptive neuromuscular facilitation (87).

Balance exercise training consists of the alignment of the body's center of gravity regarding feet. Balance training is effective in improving the postural control and prevents falls. Balance training protocols contain exercises related to activities of everyday living, walking through obstacles, proactive and reactive balance exercises on stable/unstable surfaces (88).

High Intensity Intermittent Training (HIIT) is an exercise regimen comprising multiple high- intensity training and low- intensity training intervals. In comparison to other exercise interventions, it can utilize a shorter time to achieve the same training effects characterized by brief periods, not more than 4 min of intense continuous exercise (80%–100% peak HR), interspersed with short periods of recovery or rest (89).

### 2.5 Exercise prescription for individuals with CV risk factors

#### 2.5.1 Arterial hypertension

Physical inactivity increases the risk of developing high blood pressure by 5%–13% (90). After a single session of aerobic activity, systolic blood pressure decreases by 6–12 mmHg, this is post-exercise hypotension. A systematic review and Meta-analysis have demonstrated blood pressure reduction with aerobic and resistance exercises in both normotensive and hypertensive patients (91, 92). Blood pressure reductions after dynamic resistance training were largest for prehypertensive participants −4.0 mmHg Confidence Interval (CI): −7.4 to −0.5 and −3.8 mmHg CI −5.7 to −1.9 mm Hg compared with patients with normal blood pressure or hypertension (90). The benefits of exercise in reducing blood pressure occur at any age and are independent of gender and race (93, 94).

The regulation of blood pressure is multifactorial including hemodynamic and neurohormonal mechanisms. Resting heart rate results reduced following regular exercise training and increased stroke volume have been described (95)*.* Prior research has demonstrated that arterial stiffness is significant reduced after regular exercise training programs (96) in part explained by beneficial vessel adaptations, vascular homeostasis*,* synthesis and bioavailability of nitric oxide (NO) (97). Different exercise modalities including aerobic, resistance and combined exercise training produce similar effect on endothelial function in individuals with prehypertension or hypertension (98)*.* In response to exercise, endothelial cells release vascular endothelia growth factor, angiopoietins and metalloproteinases which promote chemotaxis, endothelial cells migration, increase of vessel network and angiogenesis (99).

Arterial hypertension is defined as use of antihypertensive medication for blood pressure control or having repeated office values for resting systolic and or diastolic blood pressure of ≥140/90 mmHg, and primary arterial hypertension accounts for 95% of cases (100). The current prevalence of arterial hypertension is estimated to be 30%–45%. About 70% of adults of age 65 years or above are affected by hypertension. As population ages by 2025, the number of the hypertensive population, globally is expected to increase up to 60%.

In patients with arterial hypertension aerobic exercise is expected to reduce SBP in a range of −4.9 to −12 mmHg and −3.2 to −5.8 DBP (101). A recent meta-analysis study revealed that the optimum duration of moderate- intensity aerobic exercise such as walking, jogging and running for reducing blood pressure is 150 min/week (102). Indeed, each 30 min/week of aerobic exercise reduced SBP by 1.78 mmHg 95% CI: −2.22 to −1.33. Furthermore, a 100 min/ week of moderate-intensity aerobic exercise reduced resting heart rate by −5.77 bpm, 95% CI: −7.79 to −3.75. Beyond the traditional moderate intensity continuous training, a recent study concluded that the effects of high intensity interval training on SBP and DBP are not different (103), high intensity interval training is more effective in reducing SBP during daytime monitoring: weighted mean difference −4.14, 95% CI −6.98 to −1.30 mmHg and no significant adverse events are reported (104). Another study reported that SBP and DBP significantly decreased following isometric exercise training of 9.35 mmHg 95% CI −7.80 to −10.89 and 4.30 mmHg 95% CI = −3.01 to −5.60 respectively. Compared to leg extension and isometric handgrip exercise wall squat resulted the most effective isometric exercise training mode intervention in blood pressure control (95).

An 8-week stretching program was superior to walking in patients with stage 1 arterial hypertension or high normal blood pressure (99). A randomized clinical trial also found that multicomponent training associated with flexibility training improved blood pressure control in older inactive woman (105). A systematic umbrella review provided convincing and strong evidence of the importance of exercise in the prevention of hypertension and its protective effects in the treatment of hypertension by attenuation of CV risk factors (106). For the above indicated changes in blood pressure, it is important to reevaluate constantly hypertensive patients, since therapy might be excessive by the increase in physical activity, and a reduction in dosage and number of active principles might be required.

People with hypertension should choose exercises that help reduce blood pressure without reaching very high values during training (107). For instance, systolic blood pressure ≤220 mmHg and/or diastolic blood pressure values ≤105 mmHg are considered safe (108).

Table 3 summarizes the fundamental components of exercise prescription in patients with hypertension and evidence-based recommendations (17).

**Table 3:** Essential components of exercise prescription in patients with hypertension.

Component	Evidence Grade
*Frequency* a. Endurance exercise: 5–7/days week of moderate intensity 5–7 days/week b. Resistance exercise: 2 days or more/week, major muscle groups training c. 90–150 min/week of aerobic exercise, resistance training or combination of both d. Flexibility exercise: 2 days or more/week stretching for major muscle tendon groups	a. Class I, Level A b. Class I, Level A c. Class IIa, Level B
*Intensity* a. Endurance exercise: Moderate Intensity: 40–59% VO2R or HRR• 46–63% VO2max 64–76% HRmax• RPE 12–13 (6–20 scale) or 5–6 (0–10 scale)Vigorous Intensity:• 60–89% VO2R or HRR• 64–90% VO2max 77–95% HRmax RPE 14–17 (6–20 scale) or 7–8 (0–10 scale) b. Resistance exercise: • 50–80% 1-RM Moderate effort: can complete repetitions without excessive strain RPE 11–13 (6–20 scale) c. Flexibility exercise: stretch within limits of pain and slight discomfort	a. Class I, Level A b. Class IIa, Level B c. Class IIa, Level C
*Time* a. Endurance exercise: at least 30 min/ day. b. Resistance exercise: 2–4 sets, 8–12 repetitions at least 20 min c. Flexibility exercise: 10–30 s each stretch 2–4 repetitions about 10 min per session	a. Class I, Level A b. Class IIa, Level B c. Class IIa, Level B
*Type* a. Endurance exercise: walking, running, cycling, swimming b. Resistance exercise: free weights, resistance machines, functional body weight exercised c. Flexibility exercise: static or dynamic neuromuscular facilitations	a. Class I, Level A b. Class IIa, Level B c. Class IIa, Level B

#### 2.5.2 Dyslipidemia

Dyslipidemia is a well-established cardiovascular risk factor, which refers to lipid abnormalities consisting of either one or any combination of the following: elevated total cholesterol, low high-density lipoprotein cholesterol, elevated low-density lipoprotein cholesterol (LDL-c), and elevated triglycerides. The prevalence of dyslipidemia has been estimated to reach up to 53.7% in Europe (109). Previous meta-analyze study have reported reduced total cholesterol, reduced triglycerides and increased high density lipoprotein cholesterol level associated to exercise training (3) and exercise is essential for weight loss. Despite the high heterogeneity, weighted mean difference resulted 5.31 mg/ dL (95% CI 10.63–0.89) for triglycerides, 2.32 mg/dL (95% CI 1.16–3.87) for HDL-C, and 0.03 g/L (95% CI 0.02–0.04) for apolipoprotein A1 (3).

Prolonged aerobic exercise promotes upregulation of lipoprotein lipase activity, which enhances triglyceride hydrolysis (110). ATP-binding cassette transporter A-1 (ABCA1) in macrophages plays a crucial role in plasma high-density lipoprotein cholesterol (HDL-C) synthesis, and exercise increases ABCA1 mRNA expression (111). Additionally, PCSK9 activity is important in regulating low-density lipoprotein cholesterol (LDL-C) receptor levels, and regular daily exercise is independently associated with decreased PCSK9 levels over time (112).

In general, aerobic exercise tends to increase HDL-C and decrease triglycerides. Regular exercise consistently raises HDL cholesterol levels while helping to maintain or counterbalance increases in LDL cholesterol and triglycerides and higher activity levels correspond with greater improvements in HDL cholesterol. On the contrary aerobic exercise has little or no beneficial effect on low-density lipoprotein cholesterol (LDL-C) (113, 114). However, reducing LDL cholesterol and triglycerides requires more vigorous exercise intensity. High-intensity aerobic exercise proves particularly effective for improving overall lipid profiles, producing benefits that exceed those of moderate physical activity (114).

Table 4 summarizes the evidence-based recommendations for exercise prescription in patients with dyslipidemia (17, 114).

**Table 4:** Exercise prescription in patients with dyslipidemia.

Component	Evidence grade
*Frequency* a. Endurance exercise: 250–300 min/week organized in 5–7/days week based on caloric expenditure; for older adults ≥65 years with dyslipidemia ≥5 days/week of moderate intensity, ≥3 days/week of vigorous intensity; 3–5 days/week a combination of both b. Resistance exercise: 2–3 days/week c. Flexibility exercise: 2 days or more/week	a. Class I, Level A b. Class I, Level B c. Class IIb, Level C
*Intensity* a. Endurance exercise: Moderate Intensity: 40%–75% VO2R or HRR• 50%–75% VO2max• RPE 12–15 (6–20 scale)Vigorous Intensity: 60%–90% VO2R• RPE 16–17 (6–20 scale) b. Resistance exercise: 50%–69% of 1 repetition maximum to 70%–80% of 1 repetition maximum. For older adults ≥65 years with dyslipidemia progressive weight training 40%–50% to 60%–80% of 1 repetition maximum c. Flexibility exercise: stretch within limits of pain and slight discomfort	a. Class I, Level A b. Class I, Level B c. Class IIb, Level C
*Time* a. Endurance exercise: 30–60 min/day. For older adults ≥65 years with dyslipidemia 30–60 min/day of moderate intensity exercise; 20–30 min/ day for vigorous intensity. b. Resistance exercise: 2–4 sets, 8–12 repetitions, 2 sets 12–20 repetitions. For older adults ≥65 years with dyslipidemia progressive weight training ≥1 set of 10–15 repetitions progress to 1–3 sets of 8–12 repetitions; power training 6–10 repetitions. c. Flexibility exercise: 10–30 s each stretch 2–4 repetitions	a. Class I, Level A b. Class I, Level B c. Class IIb, Level C
*Type* a. Endurance exercise: walking, running, cycling, swimming. For older adults ≥65 years with dyslipidemia stationary cycling, aquatic exercise. b. Resistance exercise: free weights, resistance machines, functional body weight exercised c. Flexibility exercise: static, dynamic neuromuscular facilitations	a. Class I, Level A b. Class I, Level B c. Class IIb, Level C

#### 2.5.3 Overweight and obesity

Overweight and obesity have reached epidemic proportions and more than half of adults in almost all European Regions live with overweight or obesity. The highest levels of obesity and overweight are found in Eastern Europe and Mediterranean countries (115).

Exercise promotes thermogenesis, fat mass reduction, increases the browning of adipose tissue and increase of muscle mass, enhances glucose uptake in muscles and regulates lipid homeostasis in liver (116). The final results are characterized by modulation of neuro-hormonal axes, regulation of appetite, increased energy expender, and prevention of overweight and obesity. The effects of exercise on obesity are characterized by multiple interactive pathways which include up-regulation of the expression of thermogenic genes, release of myokines, modulation of adiponectin, ghrelin, leptin and insulin sensitivity (117).

A dose dependent relationship between exercise and weight loss has been described. Individuals who initially reported 60 min/ week of physical activity and increased to 134 min/week had a change in BMI of 0.4 kg/m2 across long term follow-up period (118), while, in a randomized controlled trial, aerobic exercise of 370 min/week in men and 295 min/week in women resulted in significant reduction of BMI (119). Moderate intensity exercise 150–250 min/week improves weight loss in combination with moderate diet restriction. A meta-analysis study which compared six randomized clinical trials ranging from 10 to 52 week found a 20% greater weight loss in diet plus exercise programs compared to diet-only programs and a 20% greater sustained weight loss after 1 year follow up (120). Resistance training does not affect weight loss but significantly impacts the modification in body composition related to increase in fat free mass and reduction of fat mass (84, 121). Table 5 summarized the evidence-based recommendations for exercise prescription in patients overweight or obese (17, 121).

**Table 5:** Exercise prescription in patients overweight or obese.

Component	Evidence Grade
*Frequency* a. Endurance exercise: 250–300 min/ week b. Resistance exercise: 2–3 days/week c. Flexibility exercise: 2 days or more/week	a. Class I, Level A b. Class I, Level A c. Class IIa, Level C
*Intensity* a. Endurance exercise: Moderate:40%–60%VO2 RRPE 11–13, progress to Moderate-Vigorous:50%–75% VO2R RPE 12–16 b. Resistance exercise: 60%–70% % of 1 repetition maximum c. Flexibility exercise: stretch within limits of pain and slight discomfort	a. Class I, Level A b. Class I, Level A c. Class IIa, Level C
*Time* a. Endurance exercise: 30–60 min/day. b. Resistance exercise: 2–4 set, 8–12 repetitions. c. Flexibility exercise: 10–30 s each stretch 2–4 repetitions	a. Class I, Level A b. Class I, Level A c. Class IIa, Level C
*Type* a. Endurance exercise: walking, running, cycling, swimming. b. Resistance exercise: free weights, resistance machines. c. Flexibility exercise: static, dynamic neuromuscular facilitations	a. Class I, Level A b. Class I, Level A c. Class IIa, Level C

#### 2.5.4 Exercise prescription for individuals with heart failure

Over 64 million patients worldwide are affected by Heart failure (HF), which is associated with extremely negative prognosis (122). Due to ageing process, the burden of chronic HF is expected to rise. The subtypes of HF includes HF with reduced ejection fraction (HFrEF), HF with mildly reduced ejection fraction (HFmrEF), and HF with preserved ejection fraction (HFpEF).

The therapeutic goals of exercise prescription in patients with chronic and stable HF are related to the improvement of exercise tolerance and to ameliorate the clinical features of HF (123). It should be mentioned that several mechanisms contribute to the beneficial effects of exercise training in patients with HF, including: restoration of balance between sympathetic and parasympathetic activity, and endothelial function improvement. Exercise training has been associated to reduced norepinephrine levels (124) and improved heart rate variability in older patients with HF (125). Chronic exercise training leads to increase of NO synthase production (126).

It should be mentioned that peak VO2 changes in response to supervised moderate intensity endurance exercise training in older patients with HF varies by HF subtype, with greater peak VO2 improvement in HFpEF compared to HFrEF: The proportion of patients with >5% and >10% improvement in peak VO2 resulted significantly higher in HFpEF vs. HFrEF patients (75% vs. 33.3%, *p*-value = 0.004 for >5% improvement and 66.7% vs. 29.2%, *p*-value = 0.009 for >10% improvement (127). In addition, patients with low baseline inflammatory biomarkers such as IL-6 and TNF-α showed significant improvements in peak VO2 with exercise training: 3.5 mL/kg/min improvement, while those with high inflammatory biomarkers showed no significant changes (128).

Currently, the population of HF patients with Cardiac Implantable Devices (CIDs) such as cardioverter-defibrillators (ICDs), cardiac resynchronization therapy-/defibrillators (CRT/CRT-D), permanent pacemakers (PM), leadless pacemakers and left ventricular assist devices (LVADs), has increased drastically. In this population exercise can partially reverse ventricular remodeling, restore endothelial function, and improve skeletal muscle abnormalities (129). CIDs patients should be carefully evaluated, and titration of exercise should be monitored by trained professionals. Adverse effects including shocks, anti-tachycardia pacing), device complications, arrhythmias, and deaths are low during exercise training of moderate to high intensity indicating safety and improvement of cardiopulmonary outcome (130, 131). Aerobic exercise training demonstrated an average increase in peak oxygen uptake of 2.61 mL/kg/min, ICD = 2.43, CRT = 3.2 mL/kg/min and LVADs = 2.2 mL/kg/min (107). It should be mentioned that compared to end stage HF VO2 peak was lower 14 ± 4.2 mL/kg/min vs. 11.2 ± 4.2 mL/kg/min, while ventilatory efficiency presented higher VE/VCO2 slope: 36 ± 8.3 vs. 42 ± 7.1 (131). Exercise intensity prescription in HF should ideally be anchored to CPET-derived VT rather than percentage-based methods, as VT1 and VT2 occur at highly variable percentages of peak HR (and HRR (39.0% ± 13% and 78.0% ± 13%), with percentage-based methods misclassifying intensity in up to one-third of patients (132)*.* Training targets should be: moderate-intensity continuous training at VT1 HR + 5–10 bpm progressing toward VT2 HR; HIIT work intervals at VT2 HR + 5–10 bpm (85%–95% peak HR) with recovery below VT1 HR. When CPET is unavailable, approximate VT1 at 69% peak HR or 40% HRR and VT2 at 89% peak HR or 78% HRR, though individual variation is substantial (27).

Exercise training program should be considered in stable patients with optimal therapy. Early mobilization should be initiated from the bedside, however in ICDs patients some reports suggest a waiting period of six months before starting exercise programs (131, 133). During the initial phase of exercise training, low-intensity endurance training may be considered with gradual increase of intensity to moderate and high in low risk and stable patients. The prescription for CRT continuous aerobic training may be similar to that used in HF patients, up to 60 min using various modalities 2–3 times per week at 60%–80% max HR based on pre-exercise tests (134). For ICD patients, target HR must remain ≥10–20 bpm below device detection threshold (typically 160–180 bpm) to prevent inappropriate shocks; a 6-month post-implantation waiting period is suggested though supervised earlier mobilization is generally safe CRT patients follow standard HF prescription targeting 60%–80% max HR for 20–60 min, 2–3 times weekly (134). All device patients require supervised initiation with ECG monitoring for 6–12 sessions to assess device function and hemodynamic response.

Nevertheless, considering the diverse risk profiles underlying heart failure pathophysiology, exercise training should be integrated and tailored alongside optimal antihypertensive therapy, heart rate control in atrial fibrillation, and metabolic and renal management strategies (135). Regarding training modalities in HFpEF patients, no significant difference in peak VO2 at 3 months was observed between high-intensity interval training and moderate continuous training (136).

HIIT is safe in supervised cardiac rehabilitation settings (137)*.* Key safety limits include: (1) reserve HIIT for stable patients demonstrating 2–4 weeks MICT tolerance; (2) maximum work interval intensity 90%–95% peak HR (VT2 + 10 bpm limit); (3) exclude patients with LVEF <25%, recent decompensation (<6 weeks), significant arrhythmias, or unstable angina; (4) begin with conservative work: rest ratios (1:2 or 1:3, e.g., 1 min work/2–3 min recovery) before progressing to 1:1; (5) include 10–15 min warm-up/cool-down; and (6) use low-impact modalities (cycling preferred) in older or frail patients. Common protocols alternate 3–4 min intervals at 90%–95% peak HR with 3–4 min recovery at 50%–70% peak HR, or 1-min work/1-min recovery intervals (138). Of note that 51% of HFrEF patients exercised below prescribed 90%–95% targets, indicating individual tolerance varies substantially and theoretical prescriptions may exceed achieved intensities (139).

Table 6 summarizes the evidence based (17) recommendation of exercise prescription in patients with HF.

**Table 6:** Exercise prescription in patients with heart failure.

Component	Evidence Grade
*Frequency* a. Endurance exercise: 3–7/days week, initially 2–3/days week b. Resistance exercise: 2–3 days/week b. Flexibility exercise: 2 days or more/week	a. Class I, Level A b. Class I, Level B c. Class IIa, Level B
*Intensity* a. Endurance exercise: CPET-Based (Preferred—Gold Standard):• VT1-VT2 heart rate range• Start: VT1 HR + 5–10 bpm Progress: Toward VT2 HR over weeks Target zone: Between VT1 and VT2 (moderate-intensity domain)If CPET Unavailable (Approximations):• 40%–70% VO2 reserve (VO2R)• 40%–70% Heart rate reserve (HRR) 50%–70% Peak VO2 from stress test• VT1 approximation: 69% peak HR or 40% HRR• VT2 approximation: ∼89% peak HR or ∼78% HRR; Alternative Methods: RPE 11–14 (6–20 Borg scale) or 3–5 (0–10 scale)•Talk Test: Can speak full sentences but not sing• Ventilatory frequency <30 breaths/min during exercise b. Resistance exercise: 40%–60% 1-RM initially (weeks 1–4)• Progress to 60%–80% 1-RM (weeks 5–12) RPE 11–13 (6–20 scale) initially Progress to RPE 13–15 (moderate effort) Avoid maximal effort, Valsalva maneuver c. Flexibility exercise: stretch within limits of pain and slight discomfort	a. Class I, Level A for CPET, Class I, Level B other methods b. Class I, Level B c. Class IIa, Level B
*Time* a. Endurance exercise: 150 min/ week b. Resistance exercise: 1–2 sets, up to 2–3 set, 8–15 repetitions c. Flexibility exercise: 10–30 s each stretch 2–4 repetitions about 10 min per session	a. Class I, Level A b. Class I, Level B c. Class IIa, Level B
*Type* a. Endurance exercise: walking, running, cycling, swimming, rowing continuous or high intensity interval training, arm ergometer. b. Resistance exercise: free weights, resistance machines, functional body weight exercised c. Flexibility exercise: static or dynamic	a. Class I, Level A b. Class I, Level B c. Class IIa, Level B

#### 2.5.5 Exercise prescription for individuals with type 2 diabetes mellitus

Diabetes is one of the most common chronic conditions, affecting almost 10% of the population, and remains a major public health concern (140). Thus, international guidelines recommend lifestyle modification and physical activity for the improvement of diabetes care. Indeed, exercise has shown to improve the glycemic control, reduce the risk for disabilities and ameliorate the overall survival.

The immediate effects of exercise on glucose homeostasis are characterized by increased expression of glucose transporter isoform 4 (GLUT4) and its translocation from an intracellular pool to the plasma membrane, which results in increased glucose transportation to muscle (140). In addition, exercise increases the expression of insulin receptor substrates-1and-2 and actives a variety of molecular pathways including Ca2+/calmodulin signaling, AMP-activated protein kinase and restores mitochondrial content and biogenesis (138, 141).

A recent meta-analysis study evaluated that about 36 min/week of brisk walking are necessary for an optimal control of control glycosylated hemoglobin (142). The minimal dose of physical activity needed to move from uncontrolled to controlled diabetes resulted 330 MET min/week, and the optimal dose of physical training was achieved at 1,100 MET min/week in all diabetes categories, resulting in HbA1c change ranging from −1.02% to −0.66% for severe uncontrolled diabetes, −0.64% to −0.49% for uncontrolled diabetes, −0.47% to −0.40% for controlled diabetes, and −0.38% to −0.24% for prediabetes (124). Despite the best protocol for exercise intervention in diabetic population is still not clear, high intensity interval training and resistance activity have been shown to improve the cardiorespiratory fitness and glycemic control (143, 144). Compared to controls, high intensity interval training showed significantly favorable effects on HbA1C: standardized mean difference (SMD) = − 0.74, 95% CI = − 1.35 to−0.14 (107). Resistance training significantly reduced HbA1c compared with controls weighted mean difference = –0.39, 95% CI −0.60 to −0.18 (143).

Furthermore, it seems that exercise has some anti-inflammatory and metabolism-improving effects (143).

Table 7 summarizes the evidence based (17) recommendation of exercise prescription in patients with type 2 diabetes.

**Table 7:** Exercise prescription in patients with type 2 diabetes.

Components	Evidence Grade
*Frequency* a. Endurance exercise: 5–7/days week b. Resistance exercise: 2–3 days/week c. Flexibility exercise: 2 days or more/week	a. Class I, Level A b. Class I level A c. Class IIb Level C
*Intensity* a. Endurance exercise: Moderate Intensity:40%–59% VO2R or HRR 50%–70% HRmaxRPE 12–13 (6–20 scale); Vigorous Intensity: 60%–85% VO2R or HRR 70%–85% HRmax RPE 14–16 (6–20 scale) b. Resistance exercise: 60%–80% 1-RM for muscle strength• 50%–70% 1-RM for muscle endurance, Progress from 60% to 80% over weeks RPE 13–15 (6–20 scale) c. Flexibility exercise: stretch within limits of pain and slight discomfort	a. Class I, Level A b. Class I, Level A c. Class IIb, Level C
*Time* a. Endurance exercise: 150 min/ week b. Resistance exercise: 2–4 set, 8–12 repetitions at least 20 min c. Flexibility exercise: 10–30 s each stretch 2–4 repetitions about 10 min per session	a. Class I, Level A b. Class I, Level A c. Class IIb, Level C
*Type* a. Endurance exercise: walking, running, cycling, swimming, continuous or high intensity interval training b. Resistance exercise: free weights, resistance machines, functional body weight exercised c. Flexibility exercise: static or dynamic	a. Class I, Level A b. Class I, Level A c. Class IIb, Level C

#### 2.5.6 Exercise prescription for individual with cancer

About 10 million deaths occurred in 2020 due to cancer. Cancers arise from a complex and multiple etiology including genetic, environmental and life-style factors. It has been reported that physical activity reduces the risk of cancers of breast, stomach colon, kidney, bladder, and endometrium. Limited evidence exists regarding the association between hematologic, prostate, pancreas and ovary cancers (145, 146). Furthermore, patients which exercised following a diagnosis of cancer were observed to have a lower risk of cancer mortality and experienced fewer severe adverse effects (147).

Exercise training in cancer patients modulates the inflammatory response by reducing pro-inflammatory cytokines (TNF-α and IL-6) while significantly increasing anti-inflammatory cytokines (IL-10), thereby lowering systemic inflammation and modulating oxidative stress (148). Exercise enhances synergistic antitumor activity between natural killer (NK) cells and CD8+ T cells in various cancers through multiple molecular pathways. These include CD8+ T cell recognition via the CXCL9/11-CXCR3 pathway, enhanced T cell receptor response to tumor-associated antigens, and increased CCL5 and CXCL10 levels mediated by elevated epinephrine levels. This epinephrine-driven mechanism accelerates CD8+ T cell recruitment, contributes to antitumor effects and ultimately limits tumor growth (149).

Additionally, exercise interventions including: walking, cycling, strength training, resistance training, yoga, or Tai Chi have a positive effect on health-related quality of life among cancer survivors (150). Among cancer survivors, exercise improved the quality of life and social functioning compared to controls SMD 0.99; 95% CI 0.41–1.57 SMD 0.49; 95% CI 0.11–0.87 after 6 months follow-up (150).

Despite, only 8% of cancer survivors engaged in 150 min/week of moderate to vigorous intensity exercise and about 66% of breast cancer patients were sedentary, cancer survivors should avoid inactivity. 150–300 min/week of moderate intensity or if possible 75–150 min/week of aerobic exercise is recommended. Resistance exercise should be performed on at least 2 days/week in combination with flexibility and balance training if no contraindications are present. Indeed, balance and muscle strengthening exercise had a positive multidimensional effect on patients receiving chemotherapy (151).

A pilot randomized controlled trial revealed that supervised continuous aerobic training and interval aerobic training significantly increased the cardiorespiratory fitness by 11.5% and 13% of breast cancer survivors and aerobic interval training had a greater influence on body weight and lower extremity strength (152). In patients undergoing chemotherapy exercise protocols resulted in a significant improvement in postural control (153). Recent studies support the combination of aerobic exercise and inspiratory muscle training in lung cancer (154), breast cancer (155), head and neck cancer (156) and patients undergoing hematopoietic stem cell transplantation (157). Indeed, combination of aerobic and high intensity training in patients with non- small cells lung cancer was associated with significant improvement in VO2 peak: 2.13 mL/Kg/min, 95%CI 0.06–4.20 (138). Three months of exercise training improved both respiratory muscle endurance +472 s; 95% CI, 217–728 and cycling endurance +428 s; 95% CI, 223–633 (155). These studies confirm that inspiratory muscle training is feasible and safe in patients affected by cancers.

Exercise has been demonstrated to be able to reduce same negative effect due to chemotherapy such as fatigue, lymphoedema, pulmonary disfunction and toxicity for the heart (158).

Furthermore, a meta-analysis has shown that exercise reduces fatigue and improves quality of life after radiation therapy (159).

In patients with prostate cancer two sessions per week of moderate-high intensity aerobic and resistance exercise reduced fatigue and psychological distress and improving social functioning and mental health (160).

Table 8 summarizes the evidence-based recommendations for exercise prescription in cancer patients (29, 161, 162).

**Table 8:** Recommendations for exercise prescription in cancer patients.

Component	Evidence Grade
*Frequency* a. Endurance exercise: 150–300 min/week of moderate intensity or 75–150 min/week of vigorous intensity, 3–5 days/week b. Resistance exercise: 2 days or more/week, major muscle groups training c. Flexibility exercise: 2–3days/week stretching for major muscle tendon groups d. Balance exercise: 2–3 days/week of 10 min of side walking, tandem walking, walking backward, cross-over walking, balancing on one leg. e. Respiratory muscle training: 2–3 days/week	a. Class I, Level A b. Class I, Level A c. Class IIa, Level B d. Class IIa, Level B e. Class IIb, Level B
*Intensity* a. Endurance exercise: Moderate Intensity: 40%–60% VO2R or HRR RPE 12–14 (6–20 scale) or 4–6 (0–10 scale) Vigorous (if tolerated): 60%–85% VO2R RPE 15–17 b. Resistance exercise: 60%–80% of 1 repetition maximum c. Flexibility exercise: stretch within limits of pain and slight discomfort d. Balance exercise: within limits of discomfort e. Respiratory muscle training: 30% of maximum inspiratory pressure	a. Class I, Level A b. Class I, Level A c. Class IIa, Level B d. Class IIa, Level B e. Class IIb, Level B
*Time* a. Endurance exercise: at least 30 min/ day. Start with 10–30 min and increase 10 min/ week. The intensity should also start from low and increased progressively till patient's tolerance and clinical conditions b. Resistance exercise: ≥1 set, ≥8 repetitions per set; at least 1 min of rest between sets c. Flexibility exercise: 10–30 s each stretch d. Balance exercise: 10 min	a. Class I, Level A b. Class I, Level A c. Class IIa, Level B d. Class IIb, Level B
*Type* a. Endurance exercise: walking, running, cycling, swimming b. Resistance exercise: major muscle groups c. Flexibility exercise: static stretches of major muscle tendon groups, tai chi; yoga d. Balance exercise: side walking, tandem walking, walking backward, cross-over walking, balancing on one leg e. Respiratory muscle training: 5 sets of 10 repetitions followed by 1–2 min of unloaded recovery breathing (off the device)	a. Class I, Level A b. Class I, Level A c. Class IIa, Level B d. Class IIa, Level B e. Class IIb, Level B

#### 2.5.7 Exercise prescription for frail individuals

Age-dependent cumulative decline in multiple physiological systems promotes the development of frailty, while physical activity and exercise improve the physical functioning.

Higher levels of physical activity were associated with 37% decreased odds of physical frailty and 49% for multidimensional frailty (163). Furthermore, exercise was shown to improve gait speed in frail individuals and significant increased SPPB score was also revealed (164).

Exercise addresses frailty through multiple systemic function improvements and effects on ageing related comorbidities. Key mechanisms include modulation of oxidative stress pathways, reduction of chronic inflammation, modification of immune response and restoration of mitochondrial function. It has been reported that trained individuals have fewer senescent CD4 + as well as CD8+ CD28−CD57 + cells, and a higher proportion of naive T cells (165), which are important in the development of atherosclerosis. Additionally, exercise induces an increased signaling of the transcription factor peroxisome proliferator-activated receptor γ coactivator-1α which improves mitochondrial function and reduces inflammation (166). Protein synthesis via activation of IGF-1 pathway is also modulated by exercise these molecular adaptations collectively improve muscle strength, physical function, and overall resilience in frail individuals.

Multimodal physical activity including a combination of aerobic, strengthening, balance, and flexibility significantly reduced the risk of falls related injuries by about 32%–40%. Nevertheless, the benefits of exercise programs in reducing the risk of falls were similar between older adults identified as being at high risk of falling compared to those who were at an unspecified risk (167). In addition, exercise significantly reduced the occurrence of falls in community dwelling older adults rate ratios = 0.63 (95% confidence interval 0.51–0.77. Exercise programs designed to prevent falls in older adults also seem to reduce the rate of falls leading to medical care and to prevent injuries caused by falls. A randomized controlled trial studying the effect of a 12-month intervention of walking, resistance exercise, and flexibility, found similar rates of serious and non-serious adverse events for both the intervention and control subjects (168), and other studies underly that life-threatening adverse events after exercise prescription are rare among frail individuals (169). Resistance training alone or in a multimodal training program may induce increases of maximal strength and improvement of functional capacity (170, 171). Although uncertainty exists regarding the principles of exercise prescription in frail individuals (172) a three-month home-based resistance exercise regimen in combination with protein intake resulted successful in reversing frailty: absolute risk reduction was 11.9% (CI: 0.8%–22.9%) (173). It should be emphasized that barriers to exercise prescription in frail patients are multidimensional. Frail individuals face physical and cognitive limitations, depression, inadequate socioeconomic support, and transportation constraints. In community settings, exercise should be prescribed with the same specificity as pharmacological interventions, clearly defining frequency, intensity, volume and progression based on evidence-based programs. Tailored interventions must also address social and behavioral factors to enhance motivation and adherence. Furthermore, since frail individuals often require assistance with healthcare management and activities of daily living, engaging caregivers and volunteers is essential for program success and sustainability (172).

Table 9 summarizes the evidence-based recommendations for exercise prescription in patients with frailty.

**Table 9:** Recommendations for exercise prescription in frail patients.

Component	Evidence Grade
*Frequency* a. Endurance exercise: • 3–5 days/week• Daily if very light intensity (e.g., short walks) b. Resistance exercise: ≥2 days/week (minimum) 3 days/week optimal c. Flexibility exercise: 2–3days/week stretching for major muscle tendon groups d. Balance exercise: 2–3 days/ week	a. Class I, Level A b. Class I, Level A c. Class I, Level B d. Class I, Level B
*Intensity* a. Endurance exercise: Initial (Weeks 1–4):Very light to light: 30%–40% HRR• RPE 9–11 (6–20 scale) or 2–3 (0–10 scale) “Comfortable” pace; can converse easily Progressive (Weeks 5–12): Light to moderate: 40%–60% HRR RPE 11–13 (6–20 scale) or 3–5 (0–10 scale) Slightly breathless but can still talk b. Resistance exercise: Initial (Weeks 1–4): Very light: 30%–50% 1-RM• RPE 9–11 (6–20 scale) “Light effort”; can do 12–15 reps easily Progressive (Weeks 5–12): Light to moderate: 50%–70% 1-RM• RPE 11–13 (6–20 scale) “Moderate effort”; last 2–3 reps challenging Advanced (>12 weeks, if tolerated): Moderate to high: 60%–80% 1-RM• RPE 13–15 (6–20 scale) c. Flexibility exercise: stretch within limits of pain and slight discomfort d. Balance exercise: within limits of discomfort	a. Class I, Level A b. Class I, Level A c. Class I, Level B d. Class I, Level B
*Time* a. Endurance exercise: 20–60 min/day. b. Resistance exercise: ≥ 1 set, 8 ≥repetitions per set at least 1 min of rest between sets c. Flexibility exercise: 10–30 s each stretch d. Balance exercise: 10 min	a. Class I, Level A b. Class I, Level A c. Class I, Level B d. Class I, Level B
*Type* a. Endurance exercise: walking, cycling, swimming, dancing b. Resistance exercise: major muscle groups c. Flexibility exercise: static stretches of major muscle tendon groups, tai chi; yoga d. Balance exercise: postural strategy, gait enhancement, center of gravity control	a. Class I, Level A b. Class I, Level A c. Class I, Level B d. Class I, Level B

Supplementary Tables 1, 2, summarize the main mechanisms related to exercise induced benefits and literature evidence regarding the effects of exercise in cardiovascular risk factors, chronic coronary disease, heart failure and cancers.

#### 2.5.8 Physical exercise and activity prescription adherence

Non-adherence to exercise therapy remains a significant challenge, with rates ranging from 40% to 50% among patients with chronic conditions (174). Across cancer, cardiovascular disease, and diabetes populations, the average adherence rate to physical activity interventions was 77%, with an overall dropout rate of 7.0% (16). However, structured interventions can substantially improve outcomes: supervised exercise therapy in patients with intermittent claudication increased adherence by 22% compared to unsupervised programs, resulting in measurable healthcare cost reductions (175). Patient-level factors significantly influence adherence rates, with prior physical activity history, fewer comorbidities, and higher educational attainment associated with improved compliance (176, 177). Program design features are equally critical—individualized prescriptions regarding frequency, intensity, and duration enhance participation, while single weekly sessions were associated with poor adherence even among physically active individuals (178). Furthermore, compared to patients with cognitive decline, individuals with preserved cognitive status more frequently dropped from exercise programs (179). Cognitive frail patients, incontinent or even institutionalized older adult may respond with a certain level of adherence in the case of a tailored and personalized exercise prescription (180). These findings may indicate that exercise prescription should be adjusted to the functional level and a gradual increase in frequency and intensity may motivate the adherence to physical activity.

While ventilatory thresholds provide physiologically grounded demarcations between exercise intensity domains, evidence supporting their use to guide training prescription remains predominantly confined to healthy populations, in the absence of adequately powered RCTs. Indeed, exercise intensity domains influence the biological response to VO2 max in healthy adults (181). In a population of sedentary adults, exercise training based on individual ventilatory thresholds resulted superior to standardized methods based on heart rate reserve (182). The use of thresholds for intensity markers accounts for individual metabolic characteristics and should be considered in the prescription of exercise intensity. Future research should prioritize large-scale RCTs comparing individualized, threshold-based exercise prescription against conventional intensity recommendations in diverse clinical populations, examining not only physiological adaptations but also patient-centered outcomes including functional capacity, symptom burden, quality of life, and long-term adherence.

Multidisciplinary team involvement, including physicians, nurses, and psychologists, positively impacts adherence rates, with more than 70% of studies emphasizing the necessity of comprehensive baseline participant assessment before program design (183). In part this finding may be explained by the supervision of the health status, and support, which may reduce the potential risks and increase self-efficacy (183, 184). Regular communication, social interaction may increase the adherence to exercise (183), and social support was reported as a significant prognostic factor of adherence to home-based physiotherapy (184). Regular and continuous use of technology, as ICTs (Information and Communication Technology), may be helpful to monitor the exercise program and also to provide instructions and feedback. A meta-analysis study of randomized controlled trials revealed that the adherence of patients using mobile applications for completion of cardiac rehabilitation programs was 1.4 times higher than the control group: relative risk = 1.38; CI 1.16–1.65 (185). Therefore, applications have a great potential to improve the adherence to exercise prescription the implementation of applications with intelligent wearable needs to be further explored in the terms of improvement of healthcare surveillance.

### 2.6 Digital and remote exercise delivery modalities

The integration of digital health technologies into exercise prescription has emerged as a promising strategy to overcome traditional barriers to participation, enhance adherence, and expand access to evidence-based exercise interventions in patients with chronic conditions (186).

Tele-rehabilitation combines telecommunication technologies with therapeutic exercise delivery to provide remote supervision, monitoring, and support. The 2023 American Heart Association Science Advisory on Digital Technologies in Cardiac Rehabilitation identifies telehealth as having potential to address many challenges of traditional center-based cardiac rehabilitation and augment care delivery (186).

Dual-modality programs combining synchronous telehealth exercise training through videoconferencing with asynchronous mobile health coaching apps have demonstrated feasibility in delivering guideline-concordant cardiac rehabilitation remotely. A systematic review and meta-analysis found that mobile health technology-based home cardiac rehabilitation improved adherence and achieved similar improvements in exercise capacity when compared to center-based programs (187).

Tele-rehabilitation platforms are particularly valuable for patients with transportation barriers, geographic isolation, time constraints, or medical complexity requiring expert supervision. Evidence supports application across cardiac rehabilitation, pulmonary rehabilitation, and chronic disease management programs (188).

Mobile health technology-based interventions (mHealth) have been highlighted as a promising approach for delivering home-based cardiac rehabilitation due to increasing adoption of smartphones, mobile applications, and wearable sensor devices. Recent evidence demonstrates that dual-modality programs combining synchronous telehealth exercise training through videoconferencing with asynchronous mobile health coaching apps can deliver guideline-concordant cardiac rehabilitation remotely (188, 189).

Remote or virtual cardiac rehabilitation delivery that incorporates digital technologies improves adherence and achieves similar improvements in exercise capacity when compared to in-person cardiac rehabilitation (190). Key features of effective digital cardiac rehabilitation platforms include real-time exercise monitoring with wearable sensors, remote supervision by exercise specialists, individualized progression of exercise prescription parameters, and responsive coaching based on physiological feedback. Consumer wearable activity trackers (CWATs) provide continuous monitoring of physical activity metrics including steps, heart rate, energy expenditure, and sedentary time (191, 192). Short-term interventions utilizing consumer-based wearable activity trackers generally result in increased physical activity participation (193). CWAT-based interventions effectively improve adherence to physical activity and health outcomes under free-living conditions in populations with chronic diseases. Systematic reviews demonstrate that wearable devices, when combined with behavioral multi-component strategies, can improve physical activity levels in patients with cardiovascular disease, type 2 diabetes, chronic respiratory diseases, and obesity (193). The self-management, motivational, and goal-setting properties of these commercially available devices help patients with chronic diseases better adhere to long-term physical activity in home-based settings.

Extended reality technologies, including virtual reality (VR), augmented reality (AR), and mixed reality, create immersive exercise environments that enhance engagement, motivation, and adherence through gamification and interactive feedback. A 2024 systematic review and meta-analysis of 11 studies (*n* = 1093 patients) found that virtual reality technology significantly improved exercise capacity in cardiac rehabilitation patients (mean difference: 53.26, 95% CI: 45.14–61.37) (194). VR-based cardiac rehabilitation reduced anxiety and depression scores while increasing patient engagement through immersive, multisensory experiences.

Machine learning models have demonstrated effectiveness in detecting significant patterns of physical activity behavior and associations between specific factors and intervention outcomes, with AI models generally showing higher prediction accuracy than traditional statistical approaches. Predictive algorithms can identify individuals at high risk for non-adherence, enabling proactive outreach and tailored support (195). AI models show promise but face challenges including inability to account for medication interactions, psychosocial factors, and nuanced clinical judgment required for patients with varying severity or multiple comorbidities. Current AI applications should augment, not replace, professional exercise prescription expertise.

Critical concerns in wearable application include data quality, with variation in accuracy across different metrics, devices, and populations. Healthcare providers should be aware of device limitations and validate outputs against clinical standards when making prescription decisions. Commercial wearable health devices often fall outside FDA oversight, and data not paired with doctor-patient relationships do not fall under HIPAA privacy protections, leaving much health-related metrics unregulated and potentially sold to data brokers. A 2025 systematic evaluation of privacy policies from 17 leading wearable manufacturers found high risk ratings for transparency reporting (76%) and vulnerability disclosure (65%), with significant inconsistencies in data governance across the industry (196). Digital health disparities reflect broader social determinants of health, with marginalized populations facing barriers including lack of broadband access, device ownership, digital literacy, and technology trust. Disparity populations may have unique concerns about privacy, security, and surveillance. Behavioral medicine requires application of multilevel strategies across design, delivery, dissemination, and sustainment stages to advance digital health access and foster health equity. In low-resource settings, engaging marginalized communities in planning phases is crucial, with solutions adaptable offline, scalable for limited device ownership, and addressing gender digital divide (197).

Digital exercise interventions should incorporate evidence-based behavioral change strategies including goal setting, self-monitoring, feedback, and social support. Successful implementation requires addressing digital literacy barriers and ensuring equitable access across diverse patient populations, particularly in high-risk and vulnerable groups. Healthcare providers should consider patient preferences, technological competence, and available resources when prescribing digital exercise modalities.

While digital technologies offer scalable solutions to traditional barriers such as accessibility and capacity constraints, long-term adherence strategies remain essential. Ongoing contact from healthcare professionals, whether through digital platforms or hybrid approaches combining digital and in-person interactions, enhances sustained engagement. Future research should focus on longer-term outcomes, cost-effectiveness, and strategies to promote equitable access to digital cardiac rehabilitation interventions.

## 3 Conclusions and future perspectives

In this article based on the recent international guidelines recommendation on exercise prescription we provided practical guidelines on the exercise prescription in outpatients with chronic health conditions which are parallel to the ageing process. Pre- exercise evaluation and conditions which may exclude patients from actual exercise training are also described. Caution during pre-exercise evaluation is needed in patients with organ damage secondary to hypertension (92–106), clinical features of decompensated heart failure (121–136), diabetes mellitus (137–199) and exercise intolerance in patients with frailty (161–173) and cancer diseases (145–160). Together with the tolerance to effort, specific evaluations such as muscle strength, flexibility and balance evaluations are necessary in a population with different comorbidities and disability. The principle of exercise prescription should be personalized and adopted to the current health status. A gradual and progressive incremental physical activity program is necessary to obtain the health benefits and optimize the role of exercise on outcomes. Conditions which may represent obstacles in the adherence to exercise prescription should be considered. Future studies should evaluate how social, psychological, environmental factors and technology may enhance and ameliorate the adherence to exercise prescription (174–197). The application of technology should be further implemented not only in the prescription of exercise but also during the monitoring of physical activity program.

Indeed, the use of ICTs in this context might help in engaging more the patients involved in physical activity programs (200).

It is commendable that outpatients' clinics dedicated to the prescription of physical activity in several conditions become available in health care systems. Indeed, physical activity is a modifier of clinical conditions with a similar power such as other lifestyles interventions like nutrition and diet, which are available with dedicated clinical offices and reimbursement plans to patients. In the same line, a new class of professionals, the kinesiologists, should be involved in the translation of the prescribed physical activity into training programs. Furthermore, it has been shown that routinary physical activity might play an important role in healthy ageing, as also suggested by the consensus paper from the International Exercise Recommendations in Older Adults (ICFSR) (201, 202).

These practical guidelines will assist clinicians in the selection of proper exercise training program in a safe and constructive modality.

## Definitions

- *Physical activity (PA)* is defined as any bodily movement produced by contraction of skeletal muscles that results in increased caloric requirements and energy expenditure.
- *Exercise* is a type of PA consisting in planned, structured and repetitive PA, done to maintain and improve physical fitness.
- *Physical fitness* includes the individual ability to perform activities of daily living and PA, which is important for achieving health goals.
- *FITT principles* involve frequency, intensity, time and type of exercise.
- *Endurance exercise training* defines any physical activity that uses large muscle groups and relies on aerobic metabolism to extract energy.
- *Resistance exercise training* is characterized by any physical activity that requires force against a resistance.
- *Flexibility exercise training* is the ability to move through a joint's range of motion.
- *Balance exercise training* consists of the alignment of the body's center of gravity with regard to feet
- *High Intensity Intermittent Training (HIIT)* is an exercise regimen comprising multiple high- intensity training and low- intensity training intervals.
