# Supplementary material for: Practical guidelines for exercise prescription in different clinical populations
Source: Front Sports Act Living. 2026 Mar 2;8:1649549. doi: 10.3389/fspor.2026.1649549 (PMC12990137; doi:10.3389/fspor.2026.1649549)
Supplement: Supplementary file 1 [file Supplementaryfile1.docx]

**Supplementary Table 1**. Effects of exercise in cardiovascular, metabolic, cancer and ageing comorbidities.

**List of abbreviations:** AIT: aerobic intensity training; MCT: moderate continues training; BP: blood pressure; CI: confidence interval; SBP: Systolic Blood Pressure; CNS: central nervous system; LPL : lipoprotein lipase; ABCA1: ATP-binding cassette transporter A-1; NE: norepinephrine; NT-ProBNP : terminal pro-brain natriuretic peptide; HRV: heart rate variability; CaMKs: Ca2+/calmodulin signaling and Ca2+/calmodulin-dependent protein kinases; AE: aerobic exercise; NK: natural killers;

| **First Author and Year** | **Type of Article** | **Study Design** | **Aim of study** | **Total Population** | **Main Results** |
| --- | --- | --- | --- | --- | --- |
| Ciolak E.G 2010 | Original Research | Randomized Controlled Trial | To identify effects on metabolic and hemodynamic abnormalities in normotensive offspring of hypertensive parents | 44 | AIT and MCT were equally effective in improving BP (p<0.05), insulin and insulin sensitivity (p<0.001).  AIT was superior in improving cardiorespiratory fitness (15 vs. 8%; p<0.05). |
| Edwards J.J 2022 | Systematic Review and Meta-Analysis | Meta-Analysis of Randomized Controlled Trials | Mechanisms driving BP reductions following isometric training | 628 | *Resting heart rate*  -1.55 bpm, CI = -0.14 to -2.96:  *Stroke Volume*  6.35 ml, CI = 0.35 to 12.60, *Total peripheral resistance*:  -100.38 dyne s -1 cm, CI = -14.16 to -186.61  Heart rate variability ratio  -0.41, CI = -0.09 to -0.73  *baroreceptor reflex sensitivity* 7.43 ms /mmHg, |
| Edwards J.J 2023 | Original Research | Randomized Crossover Controlled Trial | Effects of isometric training on arterial stiffness | 18 | Following isometric training early SBP : -7.7 ± 12.8 mmHg, p = 0.024),  late SBP: -5.9 ± 9.9 mmHg, p = 0.042)  DBP: -4.4 ± 7.2 mmHg, p = 0.037). |
| Pedralli M.L 2020 | Original Research | Randomized Controlled Trial | Effects of resistance training and combined training on endothelial function | 42 | Flow Mediated Dilatation  *Aerobic Training*  + 3.2% 95% CI 1.7 to 4.6  *Resistance Training*  + 4.0% 95% CI 2.1 to 5.7  *Combined Training*  +6.8% 95% CI 2.6, 11.1 |
| Ko J 2022 | Original Research | Randomized Controlled Trial | Effects of stretching vs walking exercise on population with high-normal blood pressure or stage 1 hypertension | 40 | Stretching reported greater reductions than walking program (p < .05) for sitting SBP 146 to 140 vs 139 to 142 mm Hg), supine DBP 85 to 78 vs 81 to 82 mm Hg), and nighttime diastolic 67 to 65 vs 68 to 73 mm Hg |
| Zhang Y 2024 | Review | Original Research, Reviews, Meta-analysis | To synthesizes the role of exercise as therapeutic strategy targeting obesity | NA | *CNS*  Appetite function down  Cognitive function up  Brain Structure up  Thermoregulation up  *Adipose Tissue*  Adipokines up  Thermogenesis up  *Skeletal Muscle*  Myostatin down  Irisin up  *Immune System*  IL-6 up  TLR down  TGF-B down  TNF-alfa down  *Pancreas*  Insulin up |
| Paoli A. 2015 | Review | Original Research, Reviews, Meta-analysis | To explore the metabolic effects of resistance training and its efficacy and feasibility in overweight people | NA | Thermogenesis :upregulated  Increased energy demanding for fat free mass  GH increased  Insulin reduced  Leptin, Adiponectin increased |
| Wang Y 2017 | Review | Narrative Review | To discuss the effects and mechanism of exercise training on lipid profile | NA | Exercise increases activity of LPL  and ATP-binding cassette transporter A-1 (ABCA1) in macrophages |
| Rengo G 2012 | Original Research | Prospective Cohort Study | To evaluate whether changes in plasma NE and serum NT-ProBNP terminal pro-brain natriuretic peptide after exercise training predict cardiac mortality in HF patients | 221 | Short-term changes of NE after exercise training independently predicted long-term cardiac mortality. |
| Murad K 2012 | Original Research | Randomized Controlled Trial | To explore the effect of exercise on HRV in older patients with HF | 66 | Exercise group had a significantly greater increase in both SDNN (15.46±5.02 ms in ET vs 2.37±2.13 ms in controls, p=.016) and RMSSD (17.53±7.83 ms in exercise vs 1.69±2.63 ms in controls, p=.003 |
| Stanford K 2004 | Review | Narrative Review | Mechanisms that mediate the effects of exercise to increase glucose uptake in skeletal muscle. | NA | Exercise increases skeletal muscle glucose uptake by translocation of GLUT4.  CaMKs as is a critical components of Ca2+- / exercise-stimulated skeletal muscle glucose uptake. |
| Chibalin A.V 2000 | Original Research | Experimental Model | To determine whether exercise alters the expression and activity of proteins involved in insulin-signaling in skeletal muscle. | NA | Exercise increases the expression of Insulin-stimulated receptor tyrosine phosphorylation, insulin-stimulated tyrosine phosphorylation of insulin-receptor substrate-1 and -2. |
| Sun Y. 2025 | Review | Narrative Review | To underscore the contribution of physical exercise to cancer prevention and treatment. | NA | *Inflammation*  AE helps to lower TNF-α, IL-6 and increases IL-10.  *Immune System*  Exercise enhances CD8+ T cell recognition via the CXCL9/11-CXCR3 pathway.  *Energy Metabolism*  Exercise is an effective tool for preventing and managing metabolic diseases |
| Lavin Perez AM 2023 | Review | Systematic Review and Meta-analysis of cross--sectional, case-control studies | To evaluate the effect of chronic exercise on immune cells | 462 | In women with breast cancer no significant (p > 0.05) effects of exercise interventions.  Modification of CD4 +  CD8 + NK activity. |
| Spielmann G 2011 | Original Research | Cross-Sectional Study | To evaluate the association VO2max and the age-related accumulation of senescent T-cells | 102 | VO2max was inversely associated with senescent CD4+ (B=-0.97) and CD8+ (B=-0.240) cells (p<0.05). |

**Supplementary Table 2.**

**List of abbreviations:** SBP: systolic blood pressure; DBP: diastolic blood pressure; CI: confidence interval; HIIT: high intensity interval training; MCT: moderate continues training; NA: not applicable; HF: heart failure; RT: resistance training; CIPN: Chemotherapy-induced peripheral neuropathy

| First Author and Year | Type of Article | Study Design | Aim of study | Total Population | Main Results |
| --- | --- | --- | --- | --- | --- |
| Cornelissen V.A 2013 | Review | Meta-Analysis of Randomized Controlled Trials | To investigate BP changes for different training modalities | 5223 | *Endurance*:  SBP: -3.5 mmHg  CI -4.6 to -2.3 mmHg  DBP: -2.5 mm Hg  CI-3.2 to -1.7  *Dynamic resistance*  SBP: -1.8 mm Hg  CI -3.7 to -0.011  DBP: -3.2 mm Hg  CI -4.5 to -2.0  *Isometric resistance*  SBP: -10.9 mmHg  CI-14.5 to -7.4  DBP -6.2 mm Hg  CI -10.3 to -2.0  *Combined*:  DBP: -2.2 mm Hg  CI -3.9 to -0.48  SBP: I2=0, DBP: I2=54% |
| Jabbarzadeh Ganjeh B 2024 | Review | Meta-Analysis of Randomized Controlled Trials | To evaluate the dose-dependent effects of aerobic exercise on SBP and DBP | 1787 | 150 min/week  SBP: -7.23 mmHg, 95% CI: -9.08 to -5.39  DBP: -5.58 mmHg, 95%  CI: -6.90 to -4.27 |
| Li L 2022 | Review | Meta-Analysis of Randomized Controlled Trials | To compare the effects of HIIT and MICT on BP | 422 | HIIT was better than MICT in reducing SBP during daytime monitoring  WMD = -4.14, 95% CI:  -6.98, -1.30  Heterogeneity: I2=0 % |
| Pescatello L.S 2019 | Review | Systematic review and meta-analysis | To examine and update the evidence on the relationship between physical activity and BP | 594129 | *Strong evidence*  - inverse dose-response relationship between exercise and hypertension  -physical activity reduces the risk of CVD progression among adults with hypertension  -physical activity reduces BP among adults with normal BP, prehypertension, or hypertension  -higher benefits among adults with prehypertension. |
| Lin X 2015 | Review | Meta-Analysis of Randomized Controlled Trials | To quantify the impact of exercise on cardiorespiratory fitness | 7487 | Comparing exercise groups and control groups:  *VO2 max*  0.28 L/ min 95% CI 0.23 to 0.33  3.90 mL/kg per minute 95% CI 3.45 to 4.35  *Triglycerides*  5.31 mg/ dL 95% CI 10.63 to 0.89  *HDL-C*  2.32 mg/dL 95% CI 1.16 to 3.87  *apolipoprotein A1*  0.03 g/L 95% CI 0.02 to 0.04  Interleukin-18  18.3 pg/mL; 95% CI 0.10 to 36.6  *HOMA-IR*  0.30 95% CI 0.49 to 0.11 *Hemoglobin A1c*  0.28% (95% CI 0.42 to 0.14  *Fibrinogen*  0.39 g/L 95% CI 0.03 to 0.75;  *Angiotensin II*  1.32 pg/mL 95% CI 0.54 to 2.11  I2=0% |
| Ferguson M.A 1985 | Original Research | Randomized Controlled Trial | To determine the threshold of exercise energy expenditure necessary to change blood lipid and lipoprotein concentrations | 11 | High-density lipoprotein cholesterol concentration was significantly elevated 24 h after exercise (p < 0.05) in the 1,100-, 1,300-, and 1,500-kcal sessions. |
| Man S 2013 | Review | Original Research, Reviews, Meta-analysis | To assesses the evidence from investigations and review articles that have addressed the effects of exercise on cholesterol levels. | 708 | Regular physical activity increases HDL cholesterol while maintaining, LDL cholesterol and triglycerides. A linear dose–response relationship between activity levels and HDL cholesterol levels. |
| Berk D.R 2006 | Original Research | Prospective Cohort | To study the effect of changes in physical exercise on progression of musculoskeletal disability in seniors | 549 | Inactive participants who increased exercise achieved increments in disability similar to those participants who were more active throughout.  Physical activity less than 150 min/ week will result in a nonsignificant change of weight gain compared to individuals who remain sedentary. |
| McTiernan A 2007 | Original Research | Randomized Controlled Trial | The effect of national exercise recommendations on adiposity | 202 | *Exercisers lost weight* women, -1.4 vs. +0.7 kg in controls, p = 0.008;  men, -1.8 vs. -0.1 kg in controls, p = 0.03),  *BMI*  women, -0.6 vs. +0.3 kg/m2 in controls, p = 0.006; men, -0.5 kg/m/2 vs. no change in controls, p = 0.03 |
| Curioni CC 2006 | Review | Meta-Analysis of Randomized Controlled Trials and observational studies | To assess the effectiveness of dietary interventions and exercise in long-term weight loss | 33 trials | *Diet and Exercise*  20% greater initial weight loss: 13 kg vs 9.9 kg; z=1.86-p=0.063, 95% CI. |
| Donnelley JE 2009 | Review | Update of position stand | Recommendation of physical activity intervention strategies for weight loss and prevention of weight gein | NA | *Evidence Category A.*  Physical activity prevents weight gain.  Physical activity promotes clinically significant weight loss.  Physical activity combined with energy restriction will increase weight loss.  Resistance training will not promote clinically significant weight loss.  *Evidence Category B.*  Physical activity prevents weight regain after weight loss.  Lifestyle physical activity is useful for weight management. |
| Giannuzzi P 2003 | Original Research | Randomized Trial | To study the safety and effects of exercise on LV remodeling in patients with HF | 200 | LV volumes diminished in exercise group:  (EDV, from 142+/-26 to 135+/-26 mL/m2, p<0.006; ESV, from 107+/-24 to 97+/-24 mL/m2, p<0.05)  EF improved in exercise group (p<0.001) but was unchanged in controls.  Work capacity improved (p<0.001), peak VO2 (p<0.006), walking distance (p<0.001), and QoL (p<0.01) was observed in exercise but not in controls. |
| Alswyan A.H 2018 | Review | Meta-analysis of Randomized Controlled Trials | To identify safety and efficacy of exercise training in patients with cardiac implantable devices | 5308 | *Adverse Events*  *ICD* shock events were very low 2.2% in ICD, 1.1% in VAD, and not reported in *CRT*  No lead dislodgement  *Exercise capacity*  Average increase in peak VO2 of 2.6 mL/kg/ min, (range: 2.2-3.2).  *Quality of life*  Improvement  Quality JADAD: moderate quality |
| Pistono M 2018 | Original Research | Cross-sectional | To describe the prevalence of exercise oscillatory ventilation | 192 | The occurrence of exercise oscillatory ventilation was 10%.  Mean VO2 peak consumption and elevated ventilatory response to exercise slope was lower and higher in left ventricular assist device recipients: 14 ± 4.2 ml/kg/min vs 11.2 ± 4.2 ml/kg/min; 36 ± 8.3 vs 42 ± 7.1. |
| Isaksen K 2012 | Review | Systematic Review of observational and randomized studies | To describe the effects of exercise on HF | 1889 | In 834 ICD patients seven shocks during exercise training (9.6 weeks) were registered. |
| Di R 2024 | Review | Review of guidelines, systematic reviews, meta-analyses, expert consensus, clinical decision-making, and randomized controlled trials | To retrieve, evaluate, and integrate evidence for the stratified management of motor risk in patients with CIDs | NA | Exercise risk screening assessment assessment of potential inducible ischemia and arrhythmia, evaluation of the indications of rehabilitation activities, and risk stratification of motor rehabilitation is necessary. |
| Pandey A 2017 | Original Research | Randomized Trial | To explore responses to exercise training older patients with these HFpEF vs HFrEF. | 48 | Training related improvement in peakVO2 was higher in HFpEF vs. HFrEF patients (% change: 18.7 ± 17.6 vs. −0.3 ± 15.4; p-value: <0.001). |
| Fernandez-Silva MM 2027 | Original Research | Randomized Trial | To evaluate whether inflammatory biomarkers are related to differential effect of exercise on the peak oxygen uptake VO2 among patients with HF. | 44 | Exercise significantly improved peak VO2 among participants with low inflammatory biomarkers 3.5 ± 0.9 vs. -0.7 ± 1.1 ml/kg/min per min, p = 0.006,  Exercise improved peak VO2 among participants with galectin levels below median 2.4 ± 0.8 vs. -0.3 ± 0.9 ml/kg/min p = 0.032. |
| Mueller S 2021 | Original Research | Randomized Trial | To determine whether high-intensity interval training, moderate continuous training have different effects on change in peak V̇o2 in patients with HFpEF. | 180 | HIIT vs vs guideline control <. 1.1 vs -0.6 mL/kg/min (difference, 1.5 95% CI, 0.4 to 2.7;  MCT vs guideline control, 1.6 vs -0.6 mL/kg/min (difference, 2.0 95% CI, 0.9 to 3.1;  HIIT vs MCT  1.1 vs 1.6 mL/kg/min (difference, -0.4 95% CI, -1.4 to 0.6. |
| Lazic A 2024 | Systematic Review and Meta-Analysis | Meta-analysis of clinical trials | To investigate the effects of HIIT on cardiorespiratory fitness and glycemic parameters in patients with T1DM. | 204 | *Cardiorespiratory fitness*  SMD = 0.59, 95% CI = 0.16 to 1  *24-h mean glucose control*  SMD = - 0.44, 95% CI = - 0.81 to - 0.06  *Glycated Hemoglobin* *HbA1C compared to controls*  SMD = - 0.74, 95% CI = - 1.35 to - 0.14 |
| Jansson A.K 2022 | Systematic Review and Meta-Analysis | Randomized Control Trials | To examine the effects of RT on glycemic control | 1172 | RT significantly reduced HbA1c compared with controls weighted mean difference=-0.39, 95% CI -0.60 to -0.18  No significant differences in HbA1c when comparing RT and aerobic training (p=0.42) |
| Bahar-Ozdemir Y 2020 | Original Research | Randomized Controlled Trial | To evaluate the effect of lower limb strengthening and balance exercises on balance, quality of life and neuropathic pain of the cancer patients receiving neurotoxic chemotherapy | 60 | Quality of life, physical function, and emotional status were higher, and symptom scores and PainDETECT questionnaire score were lower in the exercise group (P < 0.05) |
| Mishra SL 2012 | Review | Systematic Review and Meta-Analysis | To evaluate the effectiveness of exercise on overall HRQoL and HRQoL domains among adult post-treatment cancer survivors. | 3694 | *global HRQoL at 12 weeks'* SMD 0.48; 95% CI 0.16 to 0.81  *6 months*  SMD 0.46; 95% CI 0.09 to 0.84  *decreased anxiety at 12* *weeks'*  SMD -0.26; 95% CI -0.07 to -0.44  *fatigue at 12 weeks'*  SMD -0.82; 95% CI -1.50 to -0.14  *pain at 12 weeks*  SMD -0.29; 95% CI -0.55 to -0.04  Quality of evidence: very low to moderate. |
| Dolan LB 2016 | Original Research | Randomized Trial | To explore the safety of higher intensity exercise stimuli on cardiorespiratory fitness | 33 | *Cardiorespiratory Fitness*  improved in AIT and CMT by 12%  AIT had a greater influence on lower extremity strength (P = 0.026) and body weight (P = 0.031). |
| Duregon F 2018 | Review | Systematic Review of all evidence | To investigate the potentially beneficial effects of specific exercises to counteract the CIPN | NA | Exercise protocols for cancer patients, undergoing treatment with CIPN symptoms are feasible and effective |
| Messaggi-Sartor M 2019 | Original Research | Randomized Controlled Trial | Exercise Interventions in patients with non-small cells lung cancer | 37 | *Improvement in VO2peak* 2.13 mL/Kg/min 95% CI 0.06 to 4.20,  *Maximal inspiratory and expiratory pressures* 18.96 cmH2O 95% CI 2.7 to 24.1 and 18.58 cmH2O 95% CI 4.0 to 33.1 |
| Dahhak A 2022 | Original Research | Randomized Controlled Trial | To investigate whether inspiratory muscle training (IMT) offered adjunctively to an exercise training program reduces symptoms of dyspnea | 10 | Improved both respiratory muscle endurance (+472 seconds; 95% CI, 217-728; p=.001) and cycling endurance (+428 seconds; 95% CI, 223-633; p=.001) more than the control group. |
| Vira P 2022 | Original Research | Randomized Controlled Trial | Evaluated if a 7-week inspiratory muscle training (IMT) program during chemiotherapy is feasible, adherent, and safe. | 98 | Inspiratory Muscles Training is safe, adherent and feasable. |
| Barbosa De Almeida 2020 | Original Research | Randomized Controlled Trial | Inspiratory muscle training (IMT) program during Patients undergoing hematopoietic stem cell transplantation | 31 | IMT is safe, feasible, and improves the inspiratory muscle strength |
| Spielmann G 2011 | Original Research | Cross-sectional | The association between aerobic fitness VO2max and the age-related accumulation of senescent T-cells | 102 | T-cells and inversely associated with naïve (KLRG1-/CD28+) CD4+ (B=-1.000) and CD8+ (B=-0.993) T-cells. VO(2max) was inversely associated with senescent CD4+ (B=-0.97) and CD8+ (B=-0.240) |
| El-Khoury F 2013 | Review | Systematic review and meta-analysis of randomised controlled trials | Fall prevention exercise interventions for older community dwelling people are effective | 4305 | Exercise had a significant effect in all categories, with pooled estimates of the rate ratios of 0.63 (95% CI 0.51 to 0.77, 10 trials;  All injurious falls, 0.70 (0.54 to 0.92, 8 trials) for falls resulting in medical care, 0.57 (0.36 to 0.90, 7 trials) for severe injurious falls, and 0.39 (0.22 to 0.66, 6 trials) Heterogeneity: I2=50% |
| Life Study Investigators 2006 | Original Research | Cross-sectional | to assess the effect of a comprehensive physical activity intervention on the SPPB | 424 | The moderate intensity PA group had a lower incidence of major mobility disability defined as incapacity to complete a 400-meter walk (HR = 0.71, 95% confidence interval = 0.44-1.20). |
| Latham N.K 2004 | Original Research | Systematic review and meta-analysis of randomised controlled trials | The aim of this systematic review was to quantify the effectiveness of progressive resistance strength training to reduce physical disability | 3674 | PRT showed a strong positive effect on strength: SMD 0.68; 95% confidence interval CI 0.52, 0.84.  A modest effect was found on some measures of functional limitations such as gait speed, WMD 0.07 meters per second; 95% CI 0.04, 0.09 |
| Lopez P 2018 | Original Research | Systematic Review of randomized trials | The effect of RT alone or combined with multimodal exercise intervention on physically frailty | 371 | RT alone or in a multimodal training may induce increases of 6.6-37% in maximal strength; 3.4-7.5% in muscle mass, 8.2% in muscle power, 4.7-58.1% in functional capacity and risk of falls |
| Rizzato A 2016 | Original Research | Randomized Controlled Trials | This study aimed to assess the effectiveness of two multimodal exercise interventions (i.e., on stable and unstable surfaces) on dynamic balance control and lower limb strength in older adults. | 62 | Stable surfaces promoted faster increments of muscular strength. Unstable surfaces were more effective in enhancing dynamic balance efficiency. |
| Travers J 2023 | Original Research | Randomized Controlled Trials | To study the effectiveness of an optimised exercise and dietary protein intervention. | 168 | Combination of exercises and dietary protein significantly reduced frailty: absolute risk reduction was 11.9% (CI: 0.8%-22.9%). |
